# Laboratory- and community-based health outcomes in people with transtibial amputation using crossover and energy-storing prosthetic feet: A randomized crossover trial

**DOI:** 10.1371/journal.pone.0189652

**Published:** 2018-02-07

**Authors:** Sara J. Morgan, Cody L. McDonald, Elizabeth G. Halsne, Sarah M. Cheever, Rana Salem, Patricia A. Kramer, Brian J. Hafner

**Affiliations:** 1 Department of Rehabilitation Medicine, University of Washington, Seattle, WA, United States of America; 2 Department of Anthropology, University of Washington, Seattle, WA, United States of America; Northwestern University, UNITED STATES

## Abstract

Contemporary prosthetic feet are generally optimized for either daily or high-level activities. Prosthesis users, therefore, often require multiple prostheses to participate in activities that span a range of mobility. Crossover feet (XF) are designed to increase the range of activities that can be performed with a single prosthesis. However, little evidence exists to guide clinical prescription of XF relative to traditional energy storing feet (ESF). The objective of this study was to assess the effects of XF and ESF on health outcomes in people with transtibial amputation. A randomized crossover study was conducted to assess changes in laboratory-based (endurance, perceived exertion, walking performance) and community-based (step activity and self-reported mobility, fatigue, balance confidence, activity restrictions, and satisfaction) outcomes. Twenty-seven participants were fit with XF and ESF prostheses with standardized sockets, interfaces, and suspensions. Participants were not blinded to the intervention, and wore each prosthesis for one month while their steps were counted with an activity monitor. After each accommodation period, participants returned for data collection. Endurance and perceived exertion were measured with the Six-Minute Walk Test and Borg-CR100, respectively. Walking performance was measured using an electronic walkway. Self-reported mobility, fatigue, balance confidence, activity restrictions, and satisfaction were measured with survey instruments. Participants also reported foot preferences upon conclusion of the study. Differences between feet were assessed with a crossover analysis. While using XF, users experienced improvements in most community-based outcomes, including mobility (*p* = .001), fatigue (*p* = .001), balance confidence (*p* = .005), activity restrictions (*p* = .002), and functional satisfaction (*p* < .001). Participants also exhibited longer sound side steps in XF compared to ESF (*p* < .001). Most participants (89%) reported an overall preference for XF; others (11%) reported no preference. Results indicate that XF may be a promising alternative to ESF for people with transtibial amputation who engage in a range of mobility activities.

**Trial registration:** ClinicalTrials.gov NCT02440711

## Introduction

**Fig 1:**
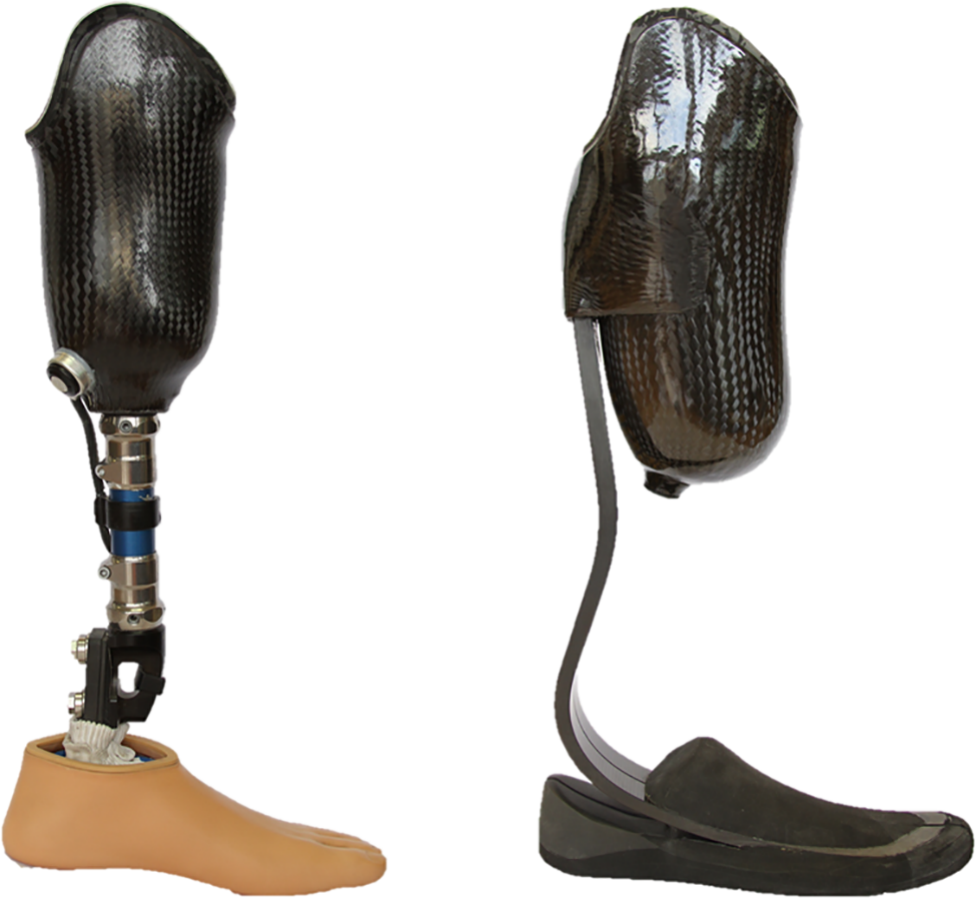
Prostheses with an ESF (left) and an XF (right).

**Fig 2:**
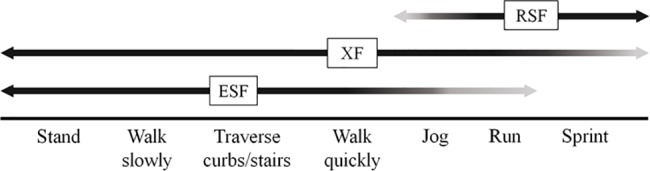
Activities along the mobility spectrum that ESF, RSF, and XF are estimated to span for most prosthetic limb users. The black sections of the arrows indicate that the foot is well-designed for these activites, the faded sections indicate that the foot design can be used for these activities, however, performance may be suboptimal.

Increasingly sophisticated prosthetic feet have been developed over the past three decades to enable users to participate in a variety of activities, including walking, running, and playing sports.[1] However, each prosthetic foot design is generally optimized for performance across a narrow range of activities and may inhibit use in other areas. For example, energy storing feet (ESF, Fig 1) are designed to both support users in static standing and facilitate their ability to walk at various speeds. ESF employ modern materials and geometric configurations designed to store and return energy in walking, much like a mechanical spring.[2] Prosthetic limbs with ESF allow users to return to an active lifestyle, but restrict running speed and aerobic performance compared to prosthetic feet designed for high speeds.[3] Running-specific feet (RSF), such as the Össur Cheetah (Össur hf, Reykjavik, Iceland), allow people with lower limb amputation to participate in demanding athletic activities.[4,5] While RSF are well-suited for running and sprinting, the absence of a heel and split keel decreases stability in standing, walking, and other low- and moderate-impact activities.[6] Thus, active users often require a primary prosthesis with an ESF and a sports prosthesis with an RSF or other specialized foot to participate optimally in a broad range of activities (Fig 2).[6,7]

Combined use of primary and sports prostheses may allow people to engage in a variety of low-, moderate-, and high-impact activities. However, obtaining and maintaining multiple prostheses may present barriers to many active prosthetic limb users. First, procuring a sports prosthesis is expensive–many insurance companies only cover the beneficiary’s primary prostheses and generally deem secondary prostheses not to be medically necessary.[8] Thus, users must often pay out-of-pocket for sports prostheses. Second, sports prostheses require regular maintenance, similar to the individual’s primary prosthesis. Prosthesis users must regularly return to their prosthetist for adjustments as components wear or the fit of their prosthesis changes over time.[6,7,9] These adjustments can be time-consuming, particularly when a second prosthesis must be modified. Another barrier to use of sports prostheses is the inconvenience of switching back and forth between prostheses as users transition among different activities. These breaks in activity are inefficient and burdensome for prosthesis users and could limit users’ ability to engage in high-impact activities. Together, the cost, maintenance, and burden of alternating prostheses may restrict participation in occasional or regular physical activity for many prosthesis users. In lieu of obtaining and maintaining multiple prostheses, users have expressed a desire for feet designed to accommodate a wider range of activities.[10]

The crossover foot (XF) is a novel prosthetic foot that has been developed to widen the range of activities a user can perform with a single prosthesis. Similar to many ESF, the XF design incorporates a split keel to accommodate uneven terrain, heel springs to facilitate heel-toe walking, and a foot shell to enable the foot to fit in a traditional shoe. However, the XF also includes elements of RSF design, such as an extended, stiff carbon keel blade that attaches directly to the posterior socket. The keel blade of the XF is longer than that of the ESF to increase the energy storage and return properties of the prosthesis (Fig 1). XF are designed for both low-impact activities, such as walking and standing, and high-impact activities, such as jogging and running (Fig 2). A foot like the XF is desirable as it improves the ability to engage in activities across the mobility spectrum without use of multiple prostheses.

At present, limited evidence is available to guide selection of XF technology. Preliminary research demonstrated that users experience modest functional benefits with XF, including better mobility at comfortable and fast speeds, improved endurance, reduced perceived exertion, and longer sound (i.e., non-prosthetic) side steps compared to the ESF conditions.[11] However, these initial findings are from a pilot study with a small sample (n = 7), short accommodation period, and non-standardized prosthetic interventions across study participants. Further, the prior study used a cross-sectional, laboratory-based design that allowed for limited assessment of participants’ experiences in the community. Additional research is therefore needed to facilitate evidence-based prescription of XF prosthetic technologies for routine daily use.

The purpose of this study was to compare a range of health outcomes for individuals with transtibial amputation in two prosthetic foot conditions: XF and ESF. A randomized crossover study was conducted to assess the relative effects of XF and ESF on laboratory-based (i.e., endurance, perceived exertion, walking performance) and community-based outcomes (i.e., step activity and self-reported mobility, fatigue, balance confidence, activity restrictions, and satisfaction). Based on preliminary evidence,[11] we hypothesized that participants would exhibit equivalent or better health outcomes when wearing XF compared to ESF.

## Materials and methods

**Fig 3:**
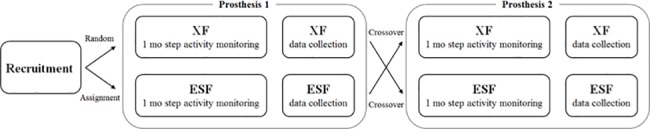
Overview of the randomized crossover study design.

A randomized crossover study (Fig 3) was conducted to compare functional and self-reported health outcomes in participants with transtibial amputation under two different test conditions: wearing a prosthesis with a ESF and wearing a prosthesis with an XF. The order in which study participants received interventions (i.e., XF-ESF or ESF-XF) was randomly assigned. Participants were not blinded to the intervention, and wore each intervention (i.e., XF and ESF) for at least one month to experience the function of each prosthetic foot across a range of settings and activities. All outcomes were assessed at the end of each one-month accommodation period.

### Participants

A convenience sample of individuals with lower limb amputation was recruited to participate in the study. An *a priori* power analysis from previously-collected data[11] was used to estimate sample size for this study. Mean differences in outcomes also proposed in the present study, including the six-minute walk test distance (mean = 19.7m, SD = 26.6m, effect size = 0.75) and walking speed (mean = 0.05m/s, SD = 0.06m/s, effect size = 0.85) were used in the power analysis. Probability of type I error (α) was set to 0.05, and power (1-β) from 0.80 to 0.95 was assessed to account for limitations in the pilot study design. G*Power 3.1.9.2 (Universität Kiel, Germany) was used to conduct the power analysis. Target sample sizes ranged from 17 to 26 based on 6MWT distances, and from 14 to 21 based on walking speeds reported in the prior study. The target sample was increased to 30 participants to account for attrition in the present study.

Study participants were recruited from local prosthetics facilities with practitioners experienced in fitting XF and ESF. Eligibility criteria included: (1) 18 years of age or older; (2) unilateral, transtibial amputation that occurred more than 1 year before recruitment into study; (3) amputation secondary to non-dysvascular causes; (4) ownership of a well-fitting, functional prosthesis with an ESF or XF; and (5) ability to complete the study protocol (e.g., willingness to wear each prosthetic foot with an activity monitor for a one-month period; ability to walk continuously for at least six minutes without assistance, read and write English, and follow verbal instructions). Potential participants were excluded from the study if they had (1) contralateral lower limb or upper limb amputation or (2) any health condition that would limit completion of the study protocol (e.g., skin breakdown, heart disease).

Blocked random allocation was used to assign participants to order of prosthetic foot interventions (i.e., XF-ESF, ESF-XF). To randomize participants to order, an investigator not involved in enrollment (BH) randomly sorted a list of 32 participant identification numbers. Additional numbers beyond the minimum sample size (n = 26) were randomized to account for attrition. The first 16 randomly sorted numbers were assigned to XF-ESF and the remaining 16 were assigned to ESF-XF. Once the orders of the interventions were assigned, the list was re-sorted in ascending order by identification number. Participants were assigned consecutive identification numbers upon enrollment along with the associated order of foot assignment. Assignment was concealed from participants and the investigators involved in enrollment until the participant was enrolled in the study.

### Experimental conditions

The intervention condition for this study was a transtibial prosthesis with an XF (Össur Cheetah Xplore®, Össur hf, Reykjavik, Iceland). The comparison condition was a transtibial prosthesis with an ESF (Össur Vari-Flex®). ESF like the Vari-Flex® are commonly prescribed for many moderate-to-high activity patients with lower limb amputation. As such, Vari-Flex® feet are a direct clinical alternative to XF.

XF and ESF prostheses were custom-fabricated for each participant by their prosthetist. Given the direct-lamination attachment of the XF to the prosthetic socket, the same socket could not be used for both conditions. Therefore, duplicate sockets were fabricated for each study participant. Each participant’s XF and ESF sockets shared a custom flexible inner socket that was transferred between the prostheses to provide equivalent socket fit and comfort between prosthetic conditions. Participants also used identical interfaces (e.g., prosthetic liners) and suspension mechanisms (e.g., elevated vacuum). Footwear was standardized between test conditions. Socket adjustments to optimize fit were allowed during the study period and were duplicated in the comparison prosthesis.

### Study variables

Laboratory- and community-based health outcomes were identified to quantify differences between the tested interventions. Outcomes were selected based on preliminary data, review of similar studies,[12] the investigators’ experience with similar patients with lower limb amputation, and clinical rationale. Laboratory-based outcomes selected included endurance, perceived exertion, and walking performance. Community-based outcomes included daily step activity and self-reported mobility, fatigue, balance confidence, activity restrictions, and satisfaction. Participants were also asked to indicate and comment on the foot they preferred for performing a variety of low-, moderate-, and high-level activities.

#### Endurance and perceived exertion

Endurance was assessed with the Six Minute Walk Test (6MWT). The 6MWT is a submaximal test of aerobic capacity and endurance that exhibits good test-retest reliability in transtibial prosthesis users.[13] This test requires the participant to walk at their fastest possible walking speed for six minutes. The test was conducted with cones separated by 30m in an unobstructed indoor hallway, as recommended in administration guidelines.[14] Encouragement was not provided during the test for either session to standardize the administration protocol.[15] The study variable associated with the 6MWT is distance (m) traveled in the six-minute period.

Perceived exertion following the 6MWT was assessed with the Borg-CR100 Rating of Perceived Exertion (RPE).[16,17] RPE has been used previously to measure exertion after the 6MWT in people with lower limb amputation.[13] The Borg RPE correlates with oxygen consumption in individuals without amputation under select situations.[18] The study variable associated with RPE is the Borg-CR100 score (range of scores is 0–120, higher scores indicate more exertion).

Participants were noted to have experienced a benefit from the XF or the ESF if they demonstrated a clinically-significant change in the 6MWT and/or the Borg CR-100. Participants were noted to have no change on these measures if they did not demonstrate clinically-significant change in either measure. A clinically-significant difference in the 6MWT was defined as a change in distance by greater than 45 meters.[19] A clinically-significant difference in the Borg was defined as a change greater than 10 points.[20]

#### Walking performance

Walking performance was assessed using a 4.9m GAITRite System (CIR Systems, Havertown, Pennsylvania). The GAITRite is a pressure-sensitive instrumented walkway that has been used extensively in the scientific literature to measure temporal-spatial parameters of gait across populations, both able-bodied[21] and impaired (including people with lower limb amputation[22–25]). It has been validated against gold-standard motion-analysis systems to provide accurate measurement of walking speed and foot placement[26] and has demonstrated good reliability[27] at both self-selected and fast speeds. Data were sampled at 80Hz as participants ambulated over the walkway during the 6MWT. The GAITRite was placed in the middle of the 30m unobstructed hallway to minimize the influence of turns, acceleration, and deceleration on collected gait data. Seven spatiotemporal variables, including average walking speed (m/s), step width (cm), prosthetic and sound side step lengths (cm), and prosthetic and sound side step times (s), were collected with the GAITRite walkway. Definitions for spatiotemporal variables are based on the GAITRite Electronic Walkway Technical Reference Manual[10] with the exception of step width, which was defined as heel-to-heel base of support.

#### Step activity

Daily step activity was measured by the StepWatch 3 step activity monitor (SAM, Modus Health LLC, Washington, DC) attached to the prosthesis. The SAM was configured to record the number of steps taken by the wearer in 1-min increments for periods of up to 60 days. The SAM has excellent evidence of measurement reliability and validity across patient populations, including persons with limb loss.[28–30] Data from the activity monitor were downloaded to a laptop computer (HP; Palo Alto, CA) at each assessment and were processed according the manufacturer's instructions using the StepWatch 3.4 Software. Step activity on test days was removed from the analysis to mitigate the influence of laboratory performance testing on participants' step activity. The study variable for step activity was mean daily step count (steps/day).

#### Mobility, fatigue, and balance confidence

Mobility was measured with the Prosthetic Limb Users Survey of Mobility (PLUS-M) version 1.2, a self-report instrument developed to be a brief, valid, and reliable measure of mobility.[31,32] PLUS-M is specific to measurement of mobility in prosthetic limb users and has been developed with data from over 1300 people with lower limb amputation. Internal consistency is greater than 0.9 and PLUS-M scores correlate with other measures of mobility in hypothesized magnitude and direction.[31] The PLUS-M was administered by a 12-item short form and computerized adaptive test; the most precise score (i.e., the score with the lowest standard error) was used in the analysis. The study variable for self-reported mobility was PLUS-M T-score (range of possible scores is 17.5–76.6, where 50 is the mean of the development sample). Higher scores indicate better mobility.

Fatigue was assessed using the Patient-Reported Outcomes Measurement Information System- Fatigue (PROMIS-F) version 1.0, a self-report questionnaire that measures symptoms and effects of fatigue on respondents’ ability to execute daily activities. PROMIS measures, including PROMIS-F, were developed using rigorous modern measurement techniques and validated in the general United States population and clinical samples.[33] PROMIS-F was administered to participants by a 12-item short form and CAT, and the most precise score (i.e., the score with the lowest standard error) was used in the analysis. The study variable for self-reported fatigue was PROMIS-F T-score (range of possible scores is 29.4–84.0, where 50 is the mean of a development sample representative of the general United States population in gender, age, race, ethnicity, and education[34]). Higher scores indicate more fatigue. Normative PROMIS-F scores in samples with lower limb amputation have been previously reported in the literature.[35]

Balance confidence was assessed with the Activities Specific Balance Confidence Scale (ABC). The ABC is a self-report measure of respondents’ confidence in performing 16 community-based standing and walking activities without falling or feeling unsteady.[36] The ABC has demonstrated good psychometric properties, including retest reliability and construct validity, in samples with lower limb amputation.[37] A revised version of the ABC with a 5-point response scale [38] was used to ease administration and improve scoring. The study variable for balance confidence was the ABC score (range of scores is 0–4, higher scores indicate more balance confidence).

#### Activity restrictions and satisfaction

The 19-item revised Trinity Amputation and Prosthesis Experience Scales is a multidimensional health instrument that measures self-reported activity restrictions (TAPES-AR) and satisfaction with the function (TAPES-FUN) and aesthetics (TAPES-AES) of a prosthesis.[39,40] The revised TAPES measures have demonstrated good internal consistency in people with lower limb amputation.[39] A revised scoring system based on subscale average scores, rather than sum scores, was adopted in consultation with the TAPES developers. The study variables for activity restriction was the TAPES-AR score, and the study variable for satisfaction was the TAPES-FUN and TAPES-AES scores (range of scores was 0–2 for each subscale, higher scores indicate more restrictions for the TAPES-AR and more satisfaction for the TAPES-FUN and TAPES-AES).

#### Prosthetic foot preference

An ad hoc exit survey was administered to participants to assess which prosthetic foot they preferred overall and which they preferred for use in a number of specific activities. Preference was measured using standardized response options (i.e., XF, ESF, no preference, not applicable). Participants were also asked to provide additional details about their experiences using each prosthesis. Development of a structured exit survey was prompted by spontaneous comments made by initial study participants. The exit survey was added to the study protocol after the study had commenced to more comprehensively document participants’ experiences with each foot. Therefore, exit survey data was collected and is presented on only a subset of sample (19 of 27 participants).

### Study protocol

All study procedures were reviewed and approved by University of Washington (approval date: 02/2015) and U.S. Army Medical Research review boards. Informed consent was obtained from all participants prior to their involvement in study activities. Enrollment for the study occurred between 05/2015 and 01/2017. Data collection occurred between 08/2015 and 05/2017.

#### Screening

Potential participants were first assessed for eligibility via a telephone screening interview. Interested individuals who met the selection criteria were invited to the laboratory for an in-person screening to verify eligibility. Investigators also inspected each candidate’s residual limb and assessed their willingness to complete study procedures. Eligible individuals completed a self-report survey that included basic demographic (e.g., age, sex) and health questions (e.g., cause of amputation, time since amputation, hours of daily prosthesis use). A research prosthetist assessed each participant’s Medicare Functional Classification Level (“K-level”)[41] via clinical inspection and interview.

#### Prosthetic fabrication and fitting

Each participant had two prostheses fabricated by their usual prosthetist. Prostheses were fabricated, fit, and aligned in accordance with good clinical practices. Optimal fit, alignment, and function of each prosthesis was verified by at least one study prosthetist (CM, EH). Participants were administered the socket comfort score[42] (SCS) at each data collection session to confirm equivalent socket fit across prosthetic conditions. A research prosthetist also affixed a SAM to each participant’s prosthetic foot, per the developers’ instructions.[28] Participants used the assigned prosthesis (i.e., XF or ESF) for a period of 1 month and returned for data collection. The other prosthesis was left with study researchers to ensure full time use of the assigned prosthesis.

#### First data collection session

Participants returned to the laboratory for testing after 1 month. The SAM was removed and the data downloaded to a laptop computer. Investigators visually reviewed the SAM data and inquired with the participant if any step activity data were missing. Participants then completed a self-report survey hosted on the Assessment Center^SM^ (Northwestern University, Chicago, IL)[43] and administered on a tablet computer (Apple iPad, Cupertino, CA). The survey included standardized instruments selected to measure participants’ mobility, fatigue, balance confidence, activity restrictions, and satisfaction. Participants were then administered the 6MWT, during which they also walked over the GAITRite walkway. Participants were familiarized with the Borg-CR100 RPE scale prior to the 6MWT and standardized instructions[14] were used in administration. Immediately upon conclusion of the 6MWT, participants reported their exertion using the Borg-CR100 RPE. Following the first data collection session, participants transitioned to the other prosthesis and used it exclusively for at least one month before returning for the second data collection session.

#### Second data collection session

All instructions and procedures for the second period of use and data collection session were identical to those described for the first period of use and data collection session. Upon conclusion of the second data collection session, participants were asked to complete the standardized exit survey. Participants were allowed to keep both prostheses at the conclusion of the study.

### Data analysis

Visual analyses (e.g., histograms) were used to evaluate distributions of the collected data. Descriptive statistics (e.g., means, standard deviations) were calculated for all measures. Differences between ESF and XF conditions were assessed based on best statistical practices for a crossover analysis.[44] Two-sample *t*-tests were first conducted to determine whether carryover effects were present. For each variable, the sum of observations (i.e., time, distance, or score, based on the selected variable) for the sequence in which participants first received ESF was compared to the sum of observations for those who first received XF. When no significant carryover effects were observed, two-sample *t*-tests were conducted to assess the effects of intervention. For each variable, the difference in observations for the sequence in which participants received ESF first was compared to the difference in observations for the sequence in which participants received XF first. Holm-Bonferroni adjustments for multiple comparisons were applied to yield an experiment-wise alpha level of .05.[45] Effect sizes (i.e., Cohen’s *d*) were computed for all measured outcomes to aid in interpretation of the results.

## Results

### Participants

**Fig 4:**
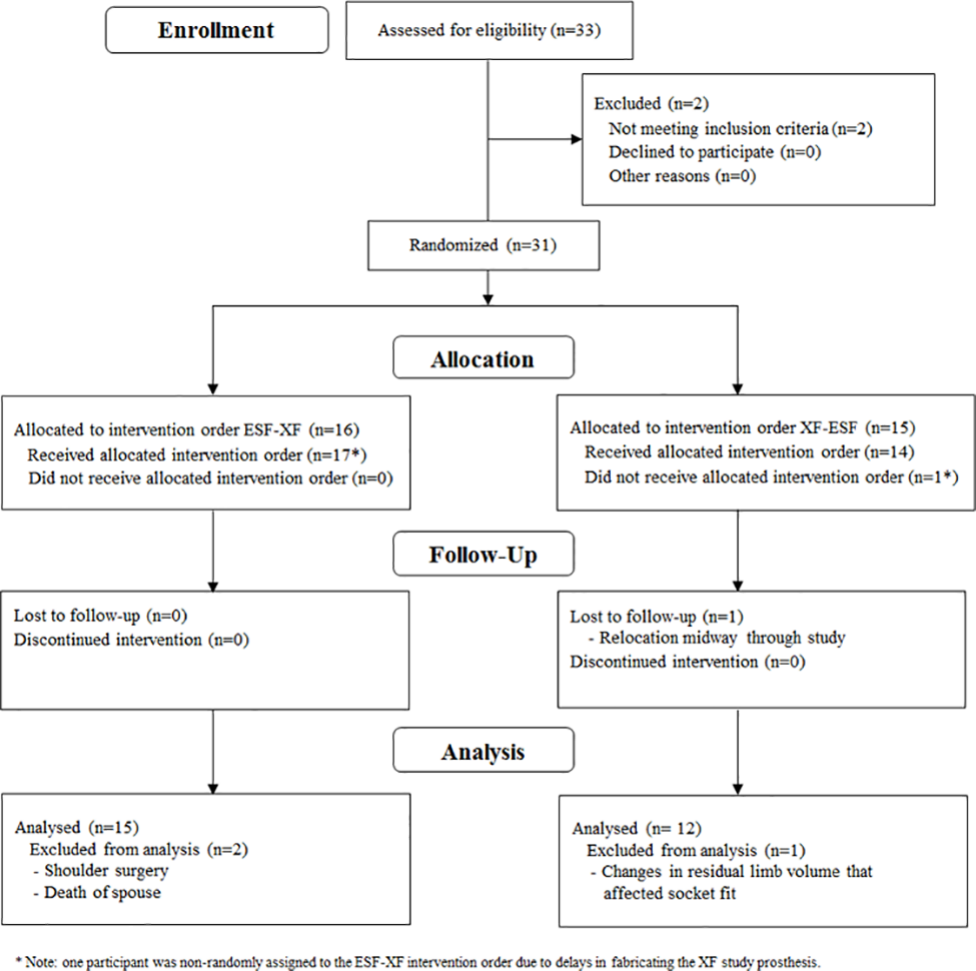
CONSORT participant flow diagram.

Thirty-one people with transtibial amputation enrolled in the study and complete datasets were collected for 30 participants. Three participants completed study protocols, but were not included in the final dataset as their health outcomes data were atypical due to shoulder surgery, significant changes in residual limb volume that affected socket fit, and the death of spouse. One participant did not complete study protocols due to relocation midway through the study (Fig 4).

Data from 27 total participants were analyzed (Table 1). All but one participant were classified by study prosthetists as unlimited community ambulators (K3, n = 16) or active adults (K4, n = 10), which is typical for people who use ESF and XF prostheses.

**Table 1:** Participant demographics and characteristics (n = 27).

Characteristic	Mean	SD
Age, years	42.3	11.0
Weight, kg	82.9	16.5
Height, cm	177.8	8.9
Time since amputation, years	11.7	10.6
Prosthesis use, hours/day	15.2	2.5
Socket Comfort Score in ESF	8.4	1.3
Socket Comfort Score in XF	8.8	1.1
	n	%
Sex, male	22	81.5%
Ethnicity		
Hispanic or Latino	1	3.7%
Not Hispanic or Latino	26	96.3%
Race		
White	23	85.2%
Black or African American	2	7.4%
American Indian or Alaskan Native	1	3.7%
Asian	0	0%
Native Hawaiian or other Pacific Islander	0	0%
Not Reported	1	3.7%
Military status		
Veteran	2	7.4%
Number comorbidities		
0	21	77.8%
1	5	18.5
2	1	3.7%
Amputation etiology		
Trauma	20	74.1%
Infection	2	7.4%
Cancer	1	3.7%
Other	4	14.8%
Medicare Functional Classification Level		
K2	1	3.7%
K3	16	59.3%
K4	10	37.0%

### Assessment of carryover effects

No significant carryover effects were observed between Session 1 and Session 2 (all *p*>.14).

### Laboratory-based outcomes

#### Endurance and perceived exertion

Participants walked similar distances (*p* = .29) during the 6MWT in the ESF and XF prostheses. Following the 6MWT, participants reported greater perceived exertion on the Borg-CR100 scale in the ESF compared to the XF condition, however this difference was not statistically significant (*p* = .05, Table 2, S1 Fig).

**Table 2:** Results of clinical performance and self-report measures (n = 27).

Outcome	ESF			XF			Difference*	Statistical test		Effect size
	Mean	SD	Range	Mean	SD	Range	XF-ESF	*t*_*25*_	*p*	*d*
Laboratory-based										
Endurance (6MWT, m)	537.1	100.8	286.8–866.5	543.3	92.6	289.2–801.5	6.2	-1.09	.29	0.06
RPE (Borg CR-100, 0–120)	47.3	28.0	0.0–100.0	36.4	31.4	0.0–100.0	-10.9	2.02	.05	-0.37
Walking Performance										
Walking speed (m/s)	1.60	0.31	0.89–2.62	1.62	0.29	0.86–2.48	0.02	-1.19	.25	0.07
Step width (cm)	13.7	3.5	7.0–24.6	13.7	3.8	7.6–25.5	0.0	0.18	.86	0.00
Prosthetic side step length (cm)	79.4	9.4	59.6–106.0	78.8	9.2	54.8–98.5	0.7	1.54	.14	-0.07
Sound side step length (cm)	76.5	9.8	43.5–94.1	79.5	10.2	45.4–95.7	3.0	-7.38	< .001	0.30
Prosthetic side step time (s)	0.49	0.05	0.38–0.57	0.49	0.05	0.38–0.55	0.0	-0.51	.61	0.00
Sound side step time (s)	0.50	0.05	0.39–0.59	0.50	0.05	0.40–0.62	0.0	-0.85	.40	0.00
Community-based										
Daily step activity (steps/day)	4307	1750	1057–8042	4109	1517	1365–6416	-198	1.54	.14	-0.12
Mobility (PLUS-M, T-score)	59.3	7.5	47.7–72.6	64.2	7.7	49.8–76.8	4.9	-3.83	.001	0.65
Fatigue (PROMIS-F, T-score)	49.1	7.4	31.7–64.3	45.4	7.5	31.7–61.3	-3.8	3.86	.001	-0.50
Balance confidence (ABC, 0–4)	3.2	0.7	1.7–4.0	3.5	0.4	2.8–4.0	0.3	-3.09	.005	0.55
Activity restrictions (TAPES-AR, 0–2)	0.6	0.4	0–1.1	0.4	0.3	0–1	-0.2	3.47	.002	-0.57
Satisfaction										
Functional (TAPES-FUN, 0–2)	1.3	0.5	0.2–2.0	1.8	0.4	0.8–2.0	0.5	-4.36	< .001	1.11
Aesthetic (TAPES-AES, 0–2)	1.5	0.5	0.7–2.0	1.5	0.5	0.7–2.0	0.0	-0.20	.85	0.00

Abbreviations: ESF = energy storing foot, XF = crossover foot, PLUS-M = Prosthetic Limb Users Survey of Mobility, PROMIS-F = Patient-reported Outcome Measurement System Fatigue, ABC = Activities-specific Balance Confidence Scale, TAPES = Trinity Amputation and Prosthesis Experience Scales, AR = Activity Restrictions, FUN = Functional Satisfaction, AES = Aesthetic Satisfaction.

*Calculated differences in XF and ESF mean values may differ due to rounding.

#### Walking performance

Participants walked with a significantly longer step length on their sound side in the XF compared to the ESF prosthesis (mean difference = 3 cm, *p* < .001). Across participants, the measured difference in step length resulted in a more symmetrical step length. Other gait parameters (i.e., walking speed, step width, step time, prosthetic side step length) collected during the 6MWT did not significantly differ between foot conditions (all *p*>.14, Table 2, S1 Fig).

### Community-based outcomes

#### Step activity

On average, participants took 198 more steps per day while wearing ESF than while wearing XF, but this difference was not statistically significant (*p* = .14, Table 2, S1 Fig).

#### Mobility, fatigue, and balance confidence

Participants reported better mobility (PLUS-M, *p* = .001), less fatigue (PROMIS-F, *p* = .001), and greater balance confidence (ABC, *p* = .005) in the XF compared to the ESF prosthesis (Table 2, S1 Fig).

#### Activity restrictions and satisfaction

Participants reported fewer activity restrictions (TAPES-AR, *p* = .002) and greater satisfaction with the function (TAPES-FUN, *p* < .001) of the XF prosthesis compared to ESF prosthesis. There was no difference in aesthetic satisfaction between conditions (TAPES-AES, *p* = .85, Table 2, S1 Fig).

### Prosthetic foot preference

Of the 19 participants that completed the standardized exit interview, 17 preferred the XF overall and two stated that they had no preference between feet. No participants preferred the ESF overall (Table 3). Activities in which the majority of participants reported a preference for the XF included walking on inclines (n = 14), ascending stairs (n = 14), walking quickly (n = 18), traversing uneven terrain (n = 11), carrying a heavy load (n = 13), playing sports (n = 12), and running (n = 15). Activities in which the majority of participants reported no preference between feet included standing up from a chair (n = 12), sitting down in a chair (n = 11), getting in and out of a car (n = 11), and walking when you cannot see your feet (n = 13). There were no activities in which the majority of participants preferred the ESF.

**Table 3:** Results of exit interview (n = 19).

	Foot Preference				Representative Participant Comments
	XF	None	ESF	N/A	
Overall	17	2	0	0	XF notes: better for high-impact activities, less fatigue, higher confidence in general, able to do more, greater propulsion, hard to adjust/align ESF notes: fits better in dress shoes/boots, doesn’t assist with propulsion (feels “flat”)
Activity					
Standing for long periods of time	7	8	3	1	Prefer XF: “more forgiving” Prefer ESF: “stability,” “feel antsy with XF”
Standing up from a chair	6	12	1	0	
Sitting down in a chair	6	11	1	1	
Getting in and out of a car	4	11	3	1	
Walking slowly	9	7	3	0	Prefer XF: “feel stable,” “more natural” Prefer ESF: “softer foot,” “doesn’t push forward”
Walking in small spaces	8	8	3	0	Prefer XF: “XF for any walking” Prefer ESF: “conducive to taking small steps”
Turning to the prosthetic side	9	8	0	2	Prefer XF: “More responsive”
Walking on inclines	14	2	3	0	Prefer XF: “more propulsion” Prefer ESF: “more toe flexibility”
Walking on declines	9	4	5	1	Prefer XF: “more control,” “better balance” Prefer ESF: “a bit more soft,” “ankle motion”
Ascending stairs	14	3	1	1	Prefer XF: “more spring return,” “lighter”
Descending stairs	7	8	4	0	Prefer XF: “feels more stable” Prefer ESF: “can control better,” “ankle motion”
Walking quickly	18	1	0	0	Prefer XF: “it’s springier, more responsive,” “smoother transition”
Walking over uneven terrain	11	5	2	1	Prefer XF: “more feeling, feels like my foot” No preference: “feels awkward either way”
Walking when you can’t see your feet	4	13	1	1	Prefer XF: “feel ground better” No preference: “dislike in general”
Dancing	9	3	1	6	Prefer XF: “feels bouncy and light”
Walking in sand	3	4	1	11	
Walking when carrying a heavy load	13	5	1	0	Prefer XF: “better balance,” “more responsive”
Playing sports	12	1	0	6	Prefer XF: “you run faster, jump higher”
Running	15	0	0	4	Prefer XF: “landing feels more gentle”

For each activity, participants were asked if they preferred the XF, had no preference, preferred the ESF, or if they had not performed the activity (N/A). Participants were also asked which foot they preferred, overall. Representative participant comments and notes were included if multiple participants made similar statements about their experiences with the XF and/or ESF. Abbreviations: ESF = energy storing foot, XF = crossover foot, N/A = not applicable

## Discussion

The goal of this study was to compare health outcomes of transtibial prosthesis users between ESF and XF prostheses. Results of this study indicate that users experienced significant improvements in most community-based health outcomes in the XF condition. Specifically, participants reported better mobility, balance confidence, and functional satisfaction and less fatigue and activity restrictions. Only one laboratory-based health outcome, sound side step length, was significantly different between foot conditions, with participants taking longer sound side steps in the XF compared to the ESF prosthesis. No significant differences were observed in endurance, rating of perceived exertion, most aspects of walking performance, daily step activity, or aesthetic satisfaction, indicating that participants experienced similar outcomes across foot conditions in these areas. Most study participants preferred the XF prosthesis overall, and particularly preferred it for performing high-level activities, such as walking when carrying a heavy load, playing sports, and running.

While overall results of this study indicate similar or improved performance in the XF relative to the ESF prosthesis, it is important to note that significant differences were based predominantly on self-report measures. Participants reported that they experienced better mobility and functional satisfaction, lower fatigue, and less activity restrictions. Interestingly, participants did not demonstrate significantly improved endurance based on 6MWT distance, significantly improved walking performance as measured by most spatiotemporal metrics, or significantly increased step activity as measured by SAMs. These findings imply people perceived benefits when wearing the XF for community-based activities, but the performance-based measures we chose for this study were unable to detect these perceived differences between feet.

Discrepancies between performance measurement and users’ experiences of outcomes related to ESF has been previously noted,[46] and is likely due to inherent differences in these approaches to health outcomes measurement. Even when assessing a singular construct, it is generally recognized that performance-based and self-report instruments measure different aspects of functioning.[47] Performance-based measures promote objectivity, but are often constrained to a laboratory-based environment and may not reflect ecologically-valid situations or settings. For example, the laboratory-based measures in the current study primarily evaluated the distance and quality of walking over a level surface. While the ability to walk long distances is important to many prosthetic limb users, such activities are not typical in daily life.[48,49] Further, walking on level surfaces may not generalize to the various activities that characterize typical home and community mobility.[50,51] In contrast, self-report measures like the PLUS-M, TAPES-AR, and ABC evaluate a variety of mobility activities beyond simple locomotion, including traversing curbs, slopes, stairs, and uneven terrain, walking while being bumped into by others, running, and participating in sports, hobbies, and vocational activities.[39,52] Laboratory-based performance measures and community-based self-report measures, therefore, provided complementary information about users’ experiences with ESF and XF in this study. Due to inherent differences in these measures, combinations of performance and self-report measures should be used in future prosthetic foot intervention research to more comprehensively assess health outcomes that are important to prosthetic foot users.

Improvements in most community-based measures, but not in laboratory-based walking over a level surface, suggests that XF may improve mobility in real-world or high-level activities. Complex mobility activities, such as those evaluated with community-based self-report measures in this study, may be more affected by prosthetic foot design than level walking, an activity that both XF and ESF are well-designed to perform. Future research is needed to assess the performance of XF and ESF in demanding, high-level mobility activities like jogging and agility tests.[53] Additionally, investigations that use direct measures of performance outside of laboratory settings are needed.

Unlike the other community-based measures used in this study, daily step activity was not significantly different between ESF and XF conditions. While step activity provides an important glimpse into locomotor activities performed by a prosthesis user in their daily life, step count has not been shown to be sensitive to interventions in recent studies that examined a variety of high- and low-activity prosthetic feet.[54–56] One possible reason for the lack of sensitivity in step count is that higher mean daily steps is not inherently better. For example, if step length is increased, as in the XF condition in this study, participants may be able to walk the same distance each day using fewer steps. Further, daily step activity is often determined by a person’s work, family, and social priorities and is, therefore, not easily varied due to the design of a prosthesis. Additionally, variations in daily step count due to atypical routine (e.g., vacation or unusual work schedule), can obscure the effects of a prosthetic foot on daily activity. More selective analysis of step activity data, guided by participants’ report of unusual activity, may provide additional insight into the relative effects of XF and ESF on community-based activity.

Sound-side step length in the current study was significantly increased in the XF compared to the ESF prosthesis (mean difference = 3.0 cm). A similar increase in sound-side step length (mean difference = 3.3 cm) was also observed for the XF in a previous cross-sectional study that assessed ESF and XF users’ walking performance.[11] Increased XF step length is further supported by participant statements in the exit interview that indicated users experienced a feeling of “more propulsion” and “smoother transition” in the XF condition. In both studies, changes in sound side step length normalized step length symmetry between the prosthetic and sound sides in the XF compared to the ESF. Improved symmetry is clinically important because it is hypothesized to reduce secondary physical conditions (e.g., osteoarthritis) common in long-term prosthesis users.[57–59]

Sound side step lengths likely increased in the XF condition due to its unique keel properties (e.g., shape and stiffness). XF keels are longer and stiffer then ESF keels, which allow more energy to be stored and returned as the keel deflects and recoils during gait. Previous research in early ESF found that the keel of the Flex Foot, which like the XF in this study had extended and posteriorly-attached keel, facilitated second rocker motion and decreased sound limb loading forces compared to other prosthetic feet (e.g., solid ankle cushion heel, SACH).[60,61] Similarly, investigation into the relationship between prosthetic keel flexibility and gait outcomes demonstrated that increased keel stiffness improved symmetry due to longer sound side steps and reduced sound side loading.[62] Thus, the increased sound-side step length observed in the present study may also decrease sound side loading due to the XF’s extended and stiff keel design. Additional research is required to better understand how the mechanical properties of prosthetic feet, including the deflection of the keel spring and the timing of the recoil, contribute to sound side kinetic outcomes and changes in users’ long-term physical health.

Measurements of endurance (6MWT) and perceived exertion (Borg-CR100) in this study were not found to significantly differ between prosthetic foot conditions. While it was hypothesized that individual participants would walk farther during and report reduced perceived exertion after the 6MWT in the XF condition, none of the participants in this study experienced improvements on *both* measures that exceeded estimates of detectable change reported in the literature (6MWT: 45m[19], Borg: 10 points[20]). However, many participants either walked farther *or* reported reduced exertion in the XF. Specifically, almost half (48%) of participants experienced a clinically-significant benefit in either 6MWT distance or Borg RPE while walking with an XF compared to approximately one-fifth (19%) of participants who experienced a clinically-significant benefit in the ESF condition. This finding suggests that almost 50% of people with transtibial amputation experience a benefit in either endurance or exertion in long-distance walking with the XF. Previous investigators have not found significant differences in 6MWT distance when comparing prosthetic feet.[55,56] However, these studies did not include or present data from the Borg to assess the tradeoff between endurance and exertion. Future prosthetic foot intervention studies should administer the Borg immediately following the 6MWT to assess potential tradeoffs between participants’ walking performance and perceived exertion.

### Strengths and limitations

Strengths of this study include the relatively large sample size of 27 participants. Prosthetic foot comparative effectiveness studies are typically much smaller, including between 3–16 people in each study.[63] In addition, participants crossed over between interventions, so each person acted as their own control. Further, data were collected longitudinally, which allowed participants one month to accommodate to each prosthetic foot condition. The extended accommodation period also allowed for the collection of community-based outcome measures to complement laboratory-based assessment of XF and ESF. Qualitative feedback in the form of exit interviews was also collected to provide context for the other measured health outcomes. Lastly, prostheses used in the study were standardized between prosthetic foot conditions and optimized by clinicians who have extensive experience with ESF fittings and have the training required for XF fittings.

Limitations of this study include the extent to which the results can be generalized to the overall population of people with lower limb amputation. While the results of this study may be generalized to people with unilateral transtibial amputation who are deemed to be community ambulators or active adults, the effects of XF on health outcomes for people with bilateral limb amputation, higher levels (e.g., transfemoral) of amputation, and lower mobility levels were not studied. Future research should assess health outcomes related to XF use in these patient groups. In addition, there is potential for bias in participant report, both in community-based self-report measures and in qualitative exit interviews. Due to the differences in overall design between XF and ESF prostheses (e.g., the extended strut and the posterior attachment of the XF), users were not blinded to interventions. Users may have reported better outcomes in the XF condition due to its novelty. Nonetheless, results for both XF and ESF conditions were similar to or better than average for measures with published transtibial prosthesis user norms (i.e., PLUS-M,[64] ABC,[65] and PROMIS-F[35]), indicating that participants in the study reported good outcomes relative to established norms in both feet. Lastly, investigators were not blinded to the intervention. Standardized methods of data collection and analysis were used to mitigate possible investigator biases, and all investigators verified they had no financial conflicts of interest related to the study.

## Conclusions

Compared to ESF, XF improved community-based health outcomes, including perceived mobility, balance confidence, fatigue, activity restrictions, and functional satisfaction, in community ambulators and active adults with unilateral transtibial amputation. Further, sound side step length increased in the XF condition, which may convey short- and long-term kinetic benefits for the sound limb. Importantly, participants in this study largely preferred XF over the ESF for daily activities, especially high-level activities like running and sports. Prosthetic limb users demonstrated similar performance on laboratory-based measures of endurance, walking performance, and step activity between foot conditions. Overall, XF are a promising advancement in prosthetic foot technology for community ambulators or active adults with unilateral transtibial amputation, although additional research is warranted to assess their effectiveness for other patient populations with lower limb amputation.

## Supporting information

S1 Fig Results figures. Graphical depiction of endurance (6MWT), perceived exertion (Borg-CR100), spatiotemporal walking performance, and community-based outcome measure results.
(DOCX)

S1 Dataset Study data and data dictionary. (XLSX)

S1 File Study protocol. (PDF)

S2 File CONSORT checklist. (DOC)
