# Supplementary material for: Laboratory- and community-based health outcomes in people with transtibial amputation using crossover and energy-storing prosthetic feet: A randomized crossover trial
Source: PLoS One. 2018 Feb 7;13(2):e0189652. doi: 10.1371/journal.pone.0189652 (PMC5802443; doi:10.1371/journal.pone.0189652)
Supplement: S1 Fig — Graphical depiction of endurance (6MWT), perceived exertion (Borg-CR100), spatiotemporal walking performance, and community-based outcome measure results. (DOCX) [file pone.0189652.s001.docx]

**S1 Fig. Results figures**

**S1 Fig 1. Endurance (6MWT) and perceived exertion (Borg-CR100) for study participants.** Means are indicated by black triangles in each plot. Pie chart depicts the percent of participants (n=27) that exhibited clinically-significant differences in either endurance (6MWT distance) or perceived exertion (Borg CR-100 rating). A clinically-significant difference in the 6MWT was defined as a change in distance by greater than 45 meters.[1] A clinically-significant difference in the Borg was defined as a change greater than 10 points.[2]


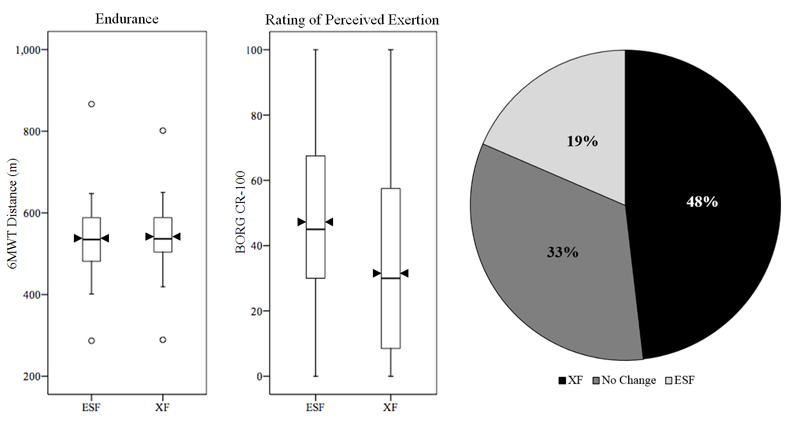


**S1 Fig 2. Spatiotemporal walking performance for study participants.** Means are indicated by black triangles in each plot.


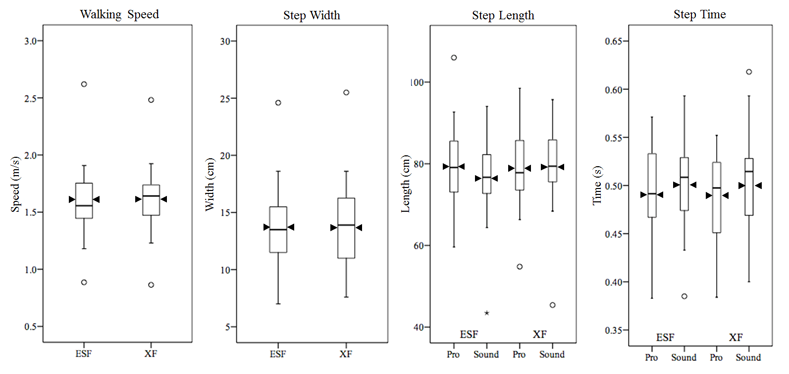


**S1 Fig 3. Community-based outcome measures.** Means are indicated by black triangles in each plot.


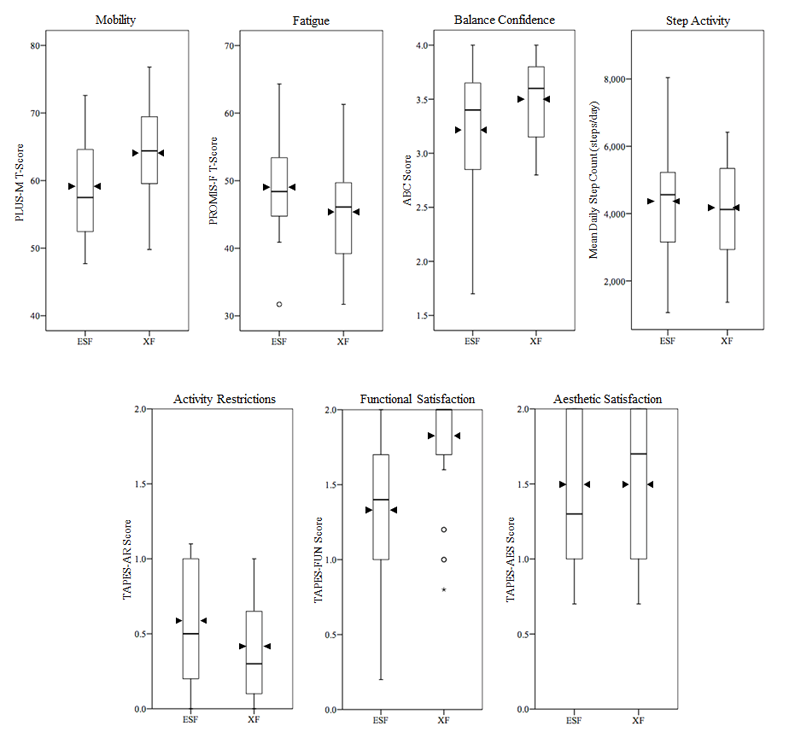


**References**

1. Resnik L, Borgia M. Reliability of outcome measures for people with lower-limb amputations: distinguishing true change from statistical error. Phys Ther. 2011;91(4):555-65.

2. Ries AL. Minimally clinically important difference for the UCSD Shortness of Breath Questionnaire, Borg Scale, and Visual Analog Scale. COPD. 2005;2(1):105-10.
