# Supplementary material for: Laboratory- and community-based health outcomes in people with transtibial amputation using crossover and energy-storing prosthetic feet: A randomized crossover trial
Source: PLoS One. 2018 Feb 7;13(2):e0189652. doi: 10.1371/journal.pone.0189652 (PMC5802443; doi:10.1371/journal.pone.0189652)
Supplement: S1 File — (PDF) [file pone.0189652.s003.pdf]

UNIVERSITY OF WASHINGTON

Human Subjects Division
Box 359470
APPLICATION: Human Subjects Review

BOX FOR COMMITTEE USE ONLY
MASTER ☐ COMM. ☐ INVESTIGATOR ☐

APPLICATION NO.

I. PRINCIPAL INVESTIGATOR (Provide all the information requested. Change of PI requires a modification. All paper-based correspondence will be directed to this person. Please list the mailing address for paper-based correspondence. You may designate a contact person other than yourself in section II., below.)

Name Brian Hafner Title PhD Position Associate Professor
Home Institution (or source of paycheck) University of Washington
UW Student? Home institution is UW.
Home UW Department (if applicable) Rehab Medicine Division Prosthetics & Orthotics
UW Position or appointment (choose the most appropriate one):

Faculty: [X] Regular Faculty Appointment [ ] Research Faculty Appointment [ ] Clinical Faculty Appointment
[ ] Visiting Faculty Appointment [ ] Dual Appointment with PNNL
[ ] Other (describe):

Student: [ ] Matriculated Undergraduate Student [ ] Graduate or Professional Student (matriculated or approved "On Leave") [ ] WWAMI Student

[ ] Resident or Fellow at the UW or Local VA [ ] UW Administration or Staff [ ] None

Campus Box # 356490 Other Address if not at UW
Telephone 206-685-4048 Fax 206-685-3244 e-mail bhafner@uw.edu

II. IRB CONTACT PERSON (Provide all the information requested. Change of Contact Person requires a modification. If this section is completed, all paper-based correspondence will be directed to this person.)

Name Cody McDonald Title CPO Position Prosthetics & Orthotics
Home Institution (or source of paycheck) University of Washington
Home UW Department (if applicable) Rehab Medicine Division Prosthetics & Orthotics
UW Position or appointment (choose the most appropriate one):

Faculty: [ ] Regular Faculty Appointment [ ] Research Faculty Appointment [ ] Clinical Faculty Appointment
[ ] Visiting Faculty Appointment [ ] Dual Appointment with PNNL
[ ] Other (describe):

Student: [ ] Matriculated Undergraduate Student [X] Graduate or Professional Student (matriculated or approved "On Leave") [ ] WWAMI Student

[ ] Resident or Fellow at the UW or Local VA [ ] UW Administration or Staff [ ] None

Campus Box # 356490 Other Address if not at UW
Telephone 206-221-6347 Fax 206-685-3244 e-mail codym@uw.edu

III. TITLE OF PROJECT: Evaluation of a Modified Running Prosthesis

IV. SIGNATURES: The undersigned acknowledge that: 1. this application is an accurate and complete description of the proposed research; 2. the research will be conducted in compliance with the recommendations of and only after approval has been received from the Institutional Review Board (IRB). The lead researcher is responsible for all aspects of this research, including: reporting any serious adverse events or problems to the IRB, requesting prior IRB approval for modifications, and requesting continuing review and approval.

A. Investigator: Brian Hafner, PhD [signature] 1/30/2015
TYPED NAME PLUS SIGNATURE **DATE**

B. Faculty sponsor (for student):
Change requires a modification.
TYPED NAME PLUS SIGNATURE **DATE**

C. The Chair, Dean, or Director acknowledges the researcher is qualified to do the research, sufficient resources will be available, and (if no external funding review occurred) there was an internal review of scientific merit.
Peter Esselman, MD 1/30/2015
TYPED NAME PLUS SIGNATURE **DATE**

____ __________ APPROVE ☐ DISAPPROVE ☐

IRB COMMITTEE SIGNATURE DATE

Subject to the following restrictions: ____

____

____

Period of approval is from_______________ through_________________

☐ Subject numbers are approved as described in this IRB Application unless otherwise indicated above in "Subject to the following conditions" or in an accompanying letter.

VALID ONLY AS LONG AS APPROVED PROCEDURES ARE FOLLOWED

V. TYPE OF NEW SUBMISSION

[ ]	**IRBshare**
[X]	**MINIMAL RISK**<br>(The research meets the **definition of minimal risk** and falls into one or more **expedited review categories**. **You are done with this table.**)
[ ]	**FULL COMMITTEE**

[ ]	Full Committee (The research involves **greater than minimal risk** and requires review at a convened meeting of the IRB)
[ ]	Full Committee (The research involves **no more than minimal risk** but does not fit into one of the expedited review categories)
[ ]	**METHODS:**

Please mark the boxes to indicate the methods which best describe your study:

[ ] **Social-Behavioral Procedures/Considerations**

[ ]	Observational	[ ]	Population-Based Field Study
[ ]	Behavioral Interventions	[ ]	Surveys/Questionnaires
[ ]	Interview/Focus Groups	[ ]	Other – Describe:

[ ] **Medical Procedures/Considerations**

[ ]	Bio-hazardous Substances	[ ]	Investigational/Approved Devices
[ ]	Controlled Substances	[ ]	Radiation Exposure
[ ]	Emergency Treatment	[ ]	Substance Abuse Treatment (with medication)
[ ]	Gene Transfer Study	[ ]	Surgical Procedures
[ ]	Stem Cell Research	[ ]	Genetic Testing
[ ]	Magnetic Resonance imaging (MRI)	[ ]	Complementary/Alternative Medicine
[ ]	Investigational/Approved Drugs and Biologics	[ ]	Other – Describe:

[ ] **DOES YOUR RESEARCH INVOLVE OR IS IT ASSOCIATED WITH ANY OF THE FOLLOWING:**

[ ]	Emergency Medicine
[ ]	Pregnant Women as a Target Population
[ ]	Genetics
[ ]	Stem Cells
[ ]	Neuroscience
[ ]	College of Arts and Sciences
[ ]	College of Education
[ ]	Dentistry
[ ]	Infectious Disease
[ ]	HIV/AIDS
[ ]	Psychiatry
[ ]	Rehabilitation Medicine
[ ]	Psycho-Social Drug Abuse Research
[ ]	Alaska Native/American Indian (ANAI)
[ ]	Public Health
[ ]	Global Health
[ ]	Health Services
[ ]	Quality of Care / Quality of Life
[ ]	Health Prevention / Health Education
[ ]	Nursing

VI. PRIMARY RESEARCH ROLES

Some research projects are conducted by a large team of individuals. Other projects can be performed by only one or two individuals. The IRB does not need to know the name of every member of your research team - instead, the IRB wants to know who is fulfilling

the following specific roles for your research. Note that the same individual may play multiple roles. If it is necessary to identify an individual by name, this will be specified below. Each section below must be completed.

1. Information for **individuals identified by name:**

The individuals below need to be identified by name. If these individuals change during the course of the research, a Modification approval from the IRB is needed before making the change.

**Subject Contact Person (to answer questions, receive complaints or reports of side effects, etc.)**			
Check here if the same as: [ ] Lead Researcher [X] IRB Contact Person<br>If one of these boxes is checked, you do not need to complete the rest of this table.			
Name ______	Title ______	Position ______	
Home Institution (or source of paycheck)<br>**UW Student? Home institution is UW.** ______			
Home UW Department (if applicable) ______	Division ______		
**UW Position or appointment (choose the most appropriate one):**			
Faculty:	[ ] Regular Faculty Appointment<br>[ ] Visiting Faculty Appointment<br>[ ] Other (describe): ______	[ ] Research Faculty Appointment<br>[ ] Dual Appointment with PNNL	[ ] Clinical Faculty Appointment
Student:	[ ] Matriculated Undergraduate Student	[ ] Graduate or Professional Student (matriculated or approved “On Leave”)	[ ] WWAMI Student
[ ] Resident or Fellow at the UW or Local VA		[ ] UW Administration or Staff	[ ] None
Campus Box # ______	Other Address if not at UW ______		
Telephone ______	Fax ______	e-mail ______	

**Study Coordinator**			
Check here if the same as: [ ] Lead Researcher [X] IRB Contact Person [ ] Subject Contact Person<br>If one of these boxes is checked, you do not need to complete the rest of this table.			
Name ______	Title ______	Position ______	
Home Institution (or source of paycheck)<br>**UW Student? Home institution is UW.** ______			
Home UW Department (if applicable) ______	Division ______		
**UW Position or appointment (choose the most appropriate one):**			
Faculty:	[ ] Regular Faculty Appointment<br>[ ] Visiting Faculty Appointment<br>[ ] Other (describe): ______	[ ] Research Faculty Appointment<br>[ ] Dual Appointment with PNNL	[ ] Clinical Faculty Appointment
Student:	[ ] Matriculated Undergraduate Student	[ ] Graduate or Professional Student (matriculated or approved “On Leave”)	[ ] WWAMI Student
[ ] Resident or Fellow at the UW or Local VA		[ ] UW Administration or Staff	[ ] None
Campus Box # ______	Other Address if not at UW ______		
Telephone ______	Fax ______	e-mail ______	

2. Information for **research staff who will perform procedures that involve risk to subjects:**

The individuals below do not need to be identified by name, rather, by qualifications. As long as the qualifications of the individuals and the procedures performed remain the same, a modification is not needed.

If an individual is **not** an agent of the UW, indicate his/her institution or organization. Should an individual not be associated with an institution or organization, state so. For all non-UW individuals, it will be necessary for this individual to receive IRB review. There are a number of mechanisms by which this may occur.

- If the non-UW individual is associated with Pacific Northwest National Laboratories, Puget Sound Blood Center, King County-Seattle Public Health, or Northwest Kidney Center: the UW has an institutional **Authorization Agreement** with these institutions by which it provides the IRB review (as long as any funding is administered through the UW).
- If the non-UW individual is associated with the Fred Hutchinson Cancer Research Center, Group Health, Seattle children’s, Swedish Medical Center, or Benaroya Research Institute at Virginia Mason: the terms of the UW’s **Cooperative IRB Agreement** with these institutions may or may not allow the UW IRB to do the review. The individual should contact their institution’s IRB for guidance.

- If the non-UW individual is associated with an institution or organization in which the UW **does not** have a Cooperative IRB Agreement, it will be necessary for the non-UW individual to provide their own IRB review. If the non-UW individual's institution or organization does not have their own IRB or does not use an IRB for review of their research and the non-UW individual's institution or organization has a Federalwide Assurance (FWA), the non-UW individual's institution or organization may enter into an **IRB Authorization Agreement** with the UW. This means that the UW will provide IRB review for the non-UW individual. The non-UW Individual's institution or organization may also wish to enter into an IRB Authorization Agreement even if they have their own IRB to prevent duplication of effort. However, entering into an IRB Authorization Agreement with a non-UW individual's institution or organization is at the discretion of the HSD.
- If the non-UW individual is not associated with an institution or organization or if the non-UW individual is associated with an institution or organization that does not have a FWA and does not routinely conduct research, an **Individual Investigator Agreement** may be entered into with the UW. The Individual Investigator Agreement extends the applicability of the UW's FWA to cover the non-UW individual, institution or organization. However, entering into an Individual Investigator Agreement with a non-UW individual, institution or organization is at the discretion of the HSD.

Please see SOP Authorization Agreements for information on how to obtain an Agreement for your research.

Study Procedures that involve risk to subjects

[ ] Phlebotomy (blood draw)

Who will perform this procedure?

[ ] Licensed Practitioner

Describe the qualifications for the Licensed Practitioner below, including whether they are affiliated with the UW, any requirement for professional license or credential, and/or experience in performing this procedure:

[ ] Study Nurse

Describe the qualifications for the Study Nurse below, including whether they are affiliated with the UW, any requirement for professional license or credential, and/or experience in performing this procedure:

[ ] Other:

Describe the qualifications for the Other Professional below, including whether they are affiliated with the UW, any requirement for professional license or credential, and/or experience in performing this procedure:

[ ] MRI Scan

Who will perform this procedure?

[ ] Licensed Practitioner

Describe the qualifications for the Licensed Practitioner below, including whether they are affiliated with the UW, any requirement for professional license or credential, and/or experience in performing this procedure:

[ ] Study Nurse

Describe the qualifications for the Study Nurse below, including whether they are affiliated with the UW, any requirement for professional license or credential, and/or experience in performing this procedure:

[ ] Other:

Describe the qualifications for the Other Professional below, including whether they are affiliated with the UW, any requirement for professional license or credential, and/or experience in performing this procedure:

[ ] Surgical or Physically Invasive Procedure

Who will perform this procedure?

[ ] Licensed Practitioner

Describe the qualifications for the Licensed Practitioner below, including whether they are affiliated with the UW, any requirement for professional license or credential, and/or experience in performing this procedure:

[ ] Study Nurse

Describe the qualifications for the Study Nurse below, including whether they are affiliated with the UW, any requirement for professional license or credential, and/or experience in performing this procedure:

[ ] Other:

Describe the qualifications for the Other Professional below, including whether they are affiliated with the UW, any requirement for professional license or credential, and/or experience in performing this procedure:

[ ] Other Procedures Involving Risk to Subjects [name the procedure(s)]:

Examples: behavioral therapy; dietary counseling; assessments and/or interpretations of test reults that require specific expertise (e.g. physical exam; fitness assessment; cognitive state; suicidality; mental health; interpretation of imaging tests, genetic tests, cognitive tests, etc.)
For more than one “Other” procedures, copy and paste this portion of the table as many times as necessary.

Who will perform this procedure?

[ ] Licensed Practitioner

Describe the qualifications of the Licensed Practitioner below, including whether they are affiliated with the UW, what is their professional license, and their experience in performing this procedure:

[ ] Study Nurse

Describe the qualifications of the Study Nurse below, including whether they are affiliated with the UW, what is their professional license, and their experience in performing this procedure:

[ ] Other:

Describe the qualifications of the Other Professional below, including whether they are affiliated with the UW, what is their professional license, and their experience in performing this procedure:

3.a. **Non-UW Individuals, Institutions or Organizations.** It will be necessary for each non-UW individual, institution or organization listed below to receive IRB review of its involvement in this research. There are a number of mechanisms by which this may occur.

Please only list the non-UW individual, institution or organization below if you are:

- **The direct recipient of an award or if you will be providing funding to the non-UW individual, institution or organization through a mechanism such as a sub-contract; and**
- **If the non-UW individual, institution or organization will be acting on behalf of the UW research study to do any of the following: 1) Obtain consent from subjects, 2) Perform procedures involving subject interaction or observation, 3) Obtain identifiable data/specimens, 4) Have access to, or receive coded or identifiable data/specimens, 5) Intervene by manipulating the environment.**
- If the non-UW individual is associated with Pacific Northwest National Laboratories, Puget Sound Blood Center, King County-Seattle Public Health, or Northwest Kidney Center: the UW has an institutional Authorization Agreement with these institutions by which it provides the IRB review (as long as any funding is administered through the UW).
- If the non-UW individual is associated with the Fred Hutchinson Cancer Research Center, Group Health, Seattle children’s, Swedish Medical Center, or Benaroya Research Institute at Virginia Mason: the terms of the UW’s **Cooperative IRB Agreement** with these institutions may or may not allow the UW IRB to do the review. The individual should contact their institution’s IRB for guidance.
- If the non-UW individual, institution or organization is one in which the UW **does not** have a Cooperative IRB Agreement, it will be necessary for the non-UW individual, institution or organization to provide their own IRB review. If the non-UW individual, institution or organization does not have their own IRB or does not use an IRB for review of their research, and the non-UW individual, institution or organization has a Federal Wide Assurance (FWA), the non-UW individual, institution or organization may enter into an **IRB Authorization Agreement** with the UW. This means that the UW will provide IRB review for the non-UW individual, institution or organization. The non-UW individual, institution or organization may also wish to enter into an IRB authorization Agreement even if they have their own IRB to prevent duplication of effort. However, entering into an IRB Authorization Agreement with a non-UW individual, institution or organization is at the discretion of the HSD.
- If the non-UW individual is not associated with an institution or organization that has a Federal Wide Assurance (FWA) or if the institution or organization listed below does not have a FWA and does not routinely conduct research, an **Individual Investigator Agreement** may be entered into with the UW. The Individual Investigator Agreement extends the applicability of the UW's FWA to cover the non-UW individual, institution or organization. However, entering into an Individual Investigator Agreement with a non-UW individual, institution or organization is at the discretion of the HSD.

Please see SOP Authorization Agreements for information on how to obtain an Agreement for your research.

3b. Non-UW Individual, Organization or Location:

If there is more than one non-UW individual, organization, or location, copy and paste this table as many times as necessary.

Name of the non-UW Individual, Organization or Location:

Address of the non-UW Individual, Organization or Location:

Describe the activities that will be performed by/at the non-UW Individual, Organization or Location (if the specified activities will not be performed, please enter N/A):	
Obtain consent from the subjects:	
Perform procedures involving subject interaction or observation:	
Obtain identifiable data/specimens?	
Have access to, or receive coded or identifiable data/specimens:	
Intervene by manipulating the environment:	

VII. SECTION 1 - LIST EACH PROPOSED AND FUNDED GRANT OR CONTRACT RELEVANT TO THIS APPLICATION, AND ATTACH A COMPLETE COPY OF EACH GRANT OR CONTRACT. THIS SHOULD INCLUDE GRANTS THAT SUPPORT FACULTY TIME FOR DATA ANALYSIS AND MANUSCRIPT PREPARATION, (I.E. SALARY SUPPORT). IF NONE, CHECK HERE ☐. FOR CENTER OR PROGRAM PROJECT GRANTS LIST P.I. AND TITLE FOR EACH SEPARATE PROJECT OR CORE. ADD SHEETS IF NECESSARY.

For Center, Program, and Institutional Training Grants (e.g., NIH “P” awards and “T” awards): Attach only the following components of the application. The terms used here are from the standard NIH applications for these types of grants. If the grant is from another agency, provide the equivalent application sections.

- Cover page(s)
- Project/Performance Site Location
- Other Project Information
- Research Plan (for Center or Program grants)
- Research Training Program Plan (for Institutional Training grants)
- Biosketch (profile) for the principal investigator on the grant

For Department of Defense (DOD) funding, complete and attach the SUPPLEMENT: Department of Defense
For Department of Justice (DOJ) funding, complete and attach the SUPPLEMENT: Department of Justice

A. Type of proposal: ☒ Research ☐ Contract ☐ Fellowship ☐ Training grant ☐ Subcontract ☐ Other, specify

B. Name of principal investigator: **Brian J. Hafner, PhD**

C. Name of funding agency: **Royalty Research Fund**

D. Agency's number (if assigned): **A97186**

E. Title of proposal: **Endurance, energy expenditure, perceived function, and satisfaction of persons with transtibial limb loss using a running-specific prosthesis modified for walking**

F. Inclusive dates: from 03/01/15 through 04/30/16

G. Status: ☒ New ☐ Competing renewal ☐ Non-competing renewal

H. Submitted through UW Office of Sponsored Programs? ☒ Yes ☐ No, (attach explanation)

VIII. SUMMARY OF ACTIVITY. Answer in spaces provided (add numbered, referenced, single-sided sheets when necessary). Do not refer to an accompanying grant or contract proposal.

A. BACKGROUND AND PURPOSE OF RESEARCH. Provide relevant background information and explain **in lay language** why this research is important and what question(s) or hypotheses this activity is designed to answer.

Amputation of a limb is a life-altering event with profound physical, psychological, and social implications. To address their functional, vocational, and recreational needs, people with lower limb amputation (LLA) are often provided with a prosthesis or artificial leg. While use of a prosthesis can allow an individual to achieve a basic level of functional mobility, absence of an anatomical foot and ankle still impairs their physical performance. As a result, people with LLA regularly exhibit decreased walking speeds, diminished endurance, and restricted ability to participate in desired life situations.

Over the past three decades, increasingly sophisticated prosthetic foot designs have been developed by the prosthetics industry to replace amputated structures in the leg. Contemporary, energy storing feet (ESF) employ advanced materials and unique geometric designs to improve walking performance and endurance of their users. Although prosthetic limbs with ESF allow people with LLA the potential to return to an active lifestyle, even the most advanced ESF do not significantly reduce the increased energy demands required for walking when compared to conventional prosthetic feet.

Commercially-available running-specific feet (RSF) like the Össur Cheetah (Össur, Reykjavik, Iceland) allow people with LLA to participate in athletic activities and sporting events. RSF provide significantly enhanced performance, compared to traditional ESF, by extending the length and increasing the stiffness of the prosthetic keel (forefoot). RSF also do not include a heel, as they are used only for running activities. Although transtibial runners with RSF exhibit endurance levels similar to non-amputees, the RSF design does not allow the biomechanical movements or provide the stability needed to use the foot for walking (over level or uneven terrain).

A Seattle-area prosthetist, Greg Davidson, currently provides many of his active LLA patients with a modified running-specific foot (mRSF). The mRSF combines the running keel (forefoot) of a RSF with the walking heel of a ESF. The mRSF can then be used for walking, running, and other routine daily activities. Mr. Davidson has fit and provided this prosthetic foot on over 100 of his patients. Preliminary feedback suggests that users experience improved overall function and high satisfaction with the device. However, empirical evidence is needed to support prescription of this prosthetic foot at other clinical facilities. The goal of this study is therefore to evaluate endurance, walking performance, mobility, and perceived exertion of transtibial prosthesis users (i.e., study participants) walking with a conventional ESF and the mRSF. Results will be compared to determine if the mRSF provides superior performance to the ESF, which is commonly prescribed to most active individuals with LLA.

Hypotheses:

1a. Participants with LLA will exhibit significantly increased endurance (as evidenced by an ability to walk significantly further in a timed walking test) wearing the mRSF compared to the ESF.

1b. Participants will report significantly reduced levels of exertion after performing a timed walking test in the mRSF, compared to the ESF.

2a. Participants will report significantly increased mobility, balance confidence, and health and significantly decreased fatigue when using the mRSF, compared to the ESF.

3a. Use of the mRSF will significantly reduce participants' net energy cost of walking (ECW) at slow, comfortable, and fast walking speeds, compared to the ESF.

B. RESEARCH PROCEDURES INVOLVED.

1. Provide a complete description of: a. the study design, and b. sequence and timing of all study procedures that will be performed, e.g., volume of blood, size of biopsy, drug administration, questionnaire, name of psychological test. Provide this information for each phase of the study (pilot, screening, intervention and follow-up). **Use lay language**. Attach study flow sheet, if available.

We will conduct a study to compare endurance, walking performance, mobility, and perceived exertion of participants with transtibial amputation wearing two different prostheses: (1) a prosthesis with a modified Running Specific Foot (mRSF), and (2) a prosthesis with a Energy Storing Foot (ESF). To maximize study resources, we will recruit participants who will be receiving from their prosthetist the mRSF prosthesis as part of their normal clinical care. We will fabricate a duplicate prosthesis with the ESF as part of this study to be used as a comparison.

*Note: Both the mRSF and ESF included in this study are devices that people with LLA use daily in their normal lives outside of this study. These feet (and other components included in the prostheses created for this study) are class I devices and **e**xempt from U.S. Food and Drug Administration premarket notification procedures.*

We will conduct a randomized crossover study to compare endurance, energy expenditure, and reported health of participants with transtibial amputation wearing prostheses with the mRSF and ESF described above. Study participants will be randomly assigned to the first foot condition (i.e. ESF or mRSF). To standardize test conditions, the participant's prosthetist (Greg Davidson) will fabricate two comparable prostheses with identical (i.e. duplicated) prosthetic sockets, interfaces, and suspension mechanisms. The same socket cannot be used for both prostheses because of the unique, direct-lamination attachment of the mRSF to the socket. The duplicate socket will be fitted with common ESF, the Ossur Vari-flex foot. We have obtained written commitments from Ossur to donate prosthetic components and from Davidson Prosthetics to donate time and materials needed to fabricate the duplicate sockets and test prostheses. Participants will be asked to wear the same athletic shoes for all sessions in order to standardized footwear between tests.

Participants will be recruited from their local prosthetist's office (Davidson Prosthetics, Federal Way, WA). Davidson Prosthetics is the sole location for recruitment as this is the only provider of the mRSF in the Northwestern United States.

Participants will be asked to come to the University of Washington Assessment and Training Laboratory (ATL) for three test sessions. Participation in the study will require participants attend three visits over the course of about three months, totaling approximately 7.5 hours.

<u>Session 1</u>: At the first session, we will screen participants, explain the study to them, and obtain informed consent (**Appendix 1**). Participants who agree will then be administered a baseline survey and perform initial speed testing. The baseline survey will include non-identifiable, medical, and demographic questions we will use to characterize the study sample (**Appendix 2**). Participants will then be asked to walk on a treadmill for 6 minutes to determine their comfortable walking speed. The treadmill is equipped with full-length handrails that allow the participant to steady him/herself as needed. A researcher will be next to the subject at all times to assist the subject if he or she were to become distressed.

Participants will be given blinded control of the treadmill speed for the first 30 seconds of testing in order to select their preferred speed. Each participant's comfortable speed will be noted and used as a reference for the metabolic testing (see Session 2 and 3, described below). Slow and fast speeds will be self-selected using an identical method (blinded control of the treadmill adjustment). Participants will be asked to perform a 6 minute practice trial at their self-selected fast speed to ensure the participant is capable of sustaining the speed without undo stress. If a participant is unable to sustain the set speed for the entire 6 minutes, he/she may reduce speed as desired, and a weighted average of the start and end fast speeds will be used for treadmill testing in Sessions 2 and 3. We will also give participants a step activity monitor to wear on their prosthesis. The activity monitor is a small, unobtrusive sensor about the size of a pager that is worn on the prosthesis and measures steps taken by the participant while they are away from the lab. Participants will be shown how to attach the activity monitor to their prosthesis by study investigators. Session 1 is expected to take about 1.5 hours.

<u>Session 2</u>: The second session will be performed after participants use the randomly-assigned ESF or mRSF prosthesis for a period of 1 month. Upon arrival at the lab, study

staff will remove the activity monitor and ask the participant to complete a self-report survey (**Appendix 2**) to evaluate their perceived performance, health, and satisfaction with their prosthesis. The survey will be administered on a tablet computer (iPad) and includes the following standardized instruments selected to measure participants’ mobility, fatigue, balance confidence, activity restrictions, and satisfaction:

- Prosthetic Limb Users Survey of Mobility – PLUS-M measures respondents’ self-reported mobility with a prosthesis. The PLUS-M was developed by the investigators in a prior study to address methodological limitations present in existing mobility measures. We will administer the PLUS-M computer adaptive test (CAT) to achieve the greatest precision of measurement and ease respondents’ burden (i.e., CAT requires participants answer fewer questions than would taking the entire survey).
- PROMIS - Fatigue – PROMIS-F measures symptoms and effects of fatigue on respondents’ ability to execute daily activities over a 1-week recall period. We will also administer PROMIS-F by CAT.
- Activities Specific Balance Confidence Scale – ABC measures respondents’ confidence in performing basic ambulatory activities. We will administer a version of the ABC recommended to ease administration and improve scoring. All 16 questions of the ABC will be administered (CAT not available).
- Trinity Amputation and Prosthesis Experience Scales - Revised – TAPES-R is a multidimensional health instrument that measures activity restrictions and satisfaction with a prosthesis. We will administer 19 TAPES-R questions (CAT not available).

We will next assess participants’ walking performance and endurance using the 6-minute walk test (6MWT). The 6MWT is a sub-maximal test of aerobic capacity and endurance that exhibits good test-retest reliability in transtibial prosthesis users. The 6MWT will be conducted using a 100ft unobstructed indoor hallway, in accordance with recommended administration guidelines. During the 6MWT, participants will walk back and forth over a pressure-sensitive instrumented mat placed in the middle of the hallway. The mat will collect outcomes associated with walking such as step length, step width, walking speed. Upon conclusion of the 6MWT, participants will self-report their exertion using the Borg Rating of Perceived Exertion (RPE). The Borg RPE measures participants’ perceived exertion after completing a standardized physical test. Note, participants will perform the 6MWT after evaluation of resting O2 consumption (see below) but before the treadmill testing so as to ensure baseline O2 consumption is not adversely affected by the 6MWT.

To assess the effects of the studied prosthetic feet on energy cost of walking, we will measure participants’ real-time metabolic energy expenditure (i.e., O2 consumption) across three walking speeds (slow, comfortable, fast). The gold standard method for determining energy consumption is measurement of the amount of oxygen a person intakes (VO2) and carbon dioxide that a person expires (VCO2). We will measure VO2 and VCO2 using a portable Cosmed K4b2 monitor. The K4b2 monitor continuously samples the participant’s expiration and inspiration using a mask that fits over their nose and mouth. Plastic tubes convey the gases to sensors that are located in a small, lightweight (1 lb) backpack system. Breath-by-breath measurements of the expired gases are stored in the system until they are uploaded by the researcher to a laptop after the experiment is completed.

Participants will be fitted with the K4b2 monitor upon conclusion of the self-report survey (described above). They will sit quietly for 5 minutes to achieve steady-state resting VO2 consumption rate (ml/min). Resting VO2 rate will be quantified as an average over the final minute of resting. Participants will then stand for 5 minutes to achieve a standing VO2 consumption rate (ml/min), again averaged over the final minute of standing.

Participants will remove the K4b2 monitor and conduct the 6MWT according to the protocol described above. After completing the Borg RPE, the participant will re-don the K4b2 monitor and sit quietly in order to return to their resting VO2 rate.

Participants will begin the treadmill testing once they have returned to their resting VO2 rate.

Participants will walk for 6 minutes on the treadmill at comfortable, slow, and fast walking speeds (as determined in Session 1). The order of the speeds will be randomized within-subjects to control for fatigue. This randomized order will be carried over into the third session so that participants are tested in the same order both times. Walking VO2 consumption rate (ml/min) will be averaged over the final 1 minute period of each activity. Participants will rest at least 5 minutes between each walking trial in order to return to their resting VO2 rate.

Session 2 will require about 3.0 hours to complete. Upon conclusion of Session 2, participants will transition to the second prosthesis (ESF or mRSF, depending upon randomization) and will be asked to use it exclusively for 1 month before returning for Session 3.

Session 3: The third session will be identical to the Session 2, but will be conducted with participants using the alternate prosthesis (e.g., ESF if they were previously tested in the mRSF).

2. Would subjects undergo these or similar procedures (medical, psychological, educational, etc.) if they were not taking part in this research? ☒ No ☐ Yes If "Yes," describe how the study procedures differ from what subjects would otherwise undergo.

The study procedures include outcome measures (survey measures, 6MWT, or Borg RPE) that a prosthetist might perform in a clinic to evaluate a patient's performance with a new prosthesis. However, the Cosmed K4b2 portable metabolic analysis system and treadmill walking are unlikely to occur in normal prosthetic clinical practice. These procedures are more likely to be conducted as part of a physical therapy treatment plan and would not be considered unusual procedures at a physical therapy clinical visit.

3. Check all of the boxes below that apply to your research:

Drug administration

☐ Administration of a drug (either FDA-approved or investigational) for research purposes to a subject-patient during general or regional anesthesia.

☐ Administration of a drug (either FDA-approved or investigational) for research purposes to a subject-patient during the 1.5 hours preceding general or regional anesthesia.

Blood lines

☐ Inserting an intravenous (central or peripheral) or intra-arterial line for research purposes in a subject-patient **during** general or regional anesthesia.

Sample collection

☐ Obtaining samples of blood, urine, or cerebrospinal fluid for research purposes while a subject-patient is under general or regional anesthesia.

☐ Obtaining a research sample from tissue or organs that would not otherwise be removed during surgery, while the subject-patient is under general or regional anesthesia.

Radio-isotopes

☐ Administration of a radio-isotope for research purposes during the 3 hours prior to anesthesia or while a subject-patient is under general or regional anesthesia.

If you checked this box, you are responsible for informing **in advance** all appropriate clinical personnel (e.g., nurses, technicians, anesthesiologists, surgeons) about the administration and use of the radio-isotope, to ensure that any personal safety issues (e.g., pregnancy) can be appropriately addressed. This is a condition of IRB approval.

Experimental devices

☐ Implantation of an experimental device while a subject-patient is under general or regional anesthesia.

Other experimental manipulations or procedures

☐ Other manipulations or procedures performed solely for research purposes while a subject-patient is under general or regional anesthesia (e.g., experimental liver dialysis, experimental brain stimulation)

None of the above

☒ None of the above apply to my research

4. If you checked any box in question #3 except "none of the above", answer the following questions:

a. Provide the name and institutional affiliation of the physician anesthesiologist who is a member of your research team or who will serve as a safety consultant about the interactions between your research procedures and the general or regional anesthesia of the subject-patients. If your procedures will be performed at a UW Medicine facility or affiliate, the anesthesiologist must be a UW faculty member.

N/A

b. If you have not yet consulted with an appropriately qualified person about this issue, describe in detail your plans to do so. The IRB will not approve your application without this consultation. If UW Department of Anesthesiology approval has been obtained, please provide the Department's letter of support.

N/A

5. Required application supplements. Complete and attach the indicated SUPPLEMENT, as appropriate

a. **SUPPLEMENT: Drugs, Biologics, Botanicals** – for research involving the use of any of the following:

- Drugs regulated by the FDA (prescription, over-the-counter, approved, or investigational)
- Biologics regulated by the FDA (prescription, over-the-counter, approved, or investigational)
- Botanicals
- Dietary Supplements

b. **SUPPLEMENT: Devices** – for research involving the use of any medical device (approved or investigational; including software used with a medical device, and including mobile medical applications).

See attached (**Appendix 3**).

c. **SUPPLEMENT: Genetic Research** – submit this supplement when your research involves genetics. **Genetic research** is defined as research involving the analysis of any of the following: DNA; RNA; chromosomes; mitochondria; any or all parts of the human genome; or biomarkers such as proteins or metabolites which may be implicated in, associated with, or cosegregated with a disorder, syndrome, condition, or predisposition to disease or behavior. Usually genetic research involves the collection and/or use of human biological specimens such as blood, skin, or other tissues, nail clippings, or hair. Genetic research may also include the construction of pedigrees ("maps" of the distribution of a particular trait or condition among related individuals) or family medical histories.

d. **SUPPLEMENT: Department of Defense** – for research involving any component of the federal Department of Defense (DOD). "Involvement" means funding; collaboration or cooperative arrangements; use of facilities, resources, or personnel; use of military or civilian members of the DOD (or their records/specimens) as subjects.

e. **SUPPLEMENT: Department of Justice** – for research involving the federal Department of Justice (DOJ) or any of its components (such as the National Institute of Justice, or any facilities/personnel of the Bureau of Prisons). "Involvement" means funding; collaboration or cooperative arrangements; use of facilities, resources, or personnel; use of records or specimens from DOJ employees or from prisoners in any Bureau of Prisons facility.

f. **SUPPLEMENT: GWAS dbGaP** – for research that will involve submitting data to the federal Database of Genotyped and Phenotyped (dbGaP) information.

g. For research involving the **Department of Energy (DOE)**, researchers should consult the **CHECKLIST Department of Energy** to ensure that they have addressed all DOE requirements. However, the Checklist does not need to be completed and submitted unless the researcher believes it would be a useful attachment.

C. DECEPTION: If any deception or withholding of complete information is required for this activity, explain why this is necessary and attach a protocol explaining if, how, when, and by whom subjects will be debriefed.

No deception is required for this study.

D. SUBJECTS

The IRB reviews the number of subjects you plan to study in the context of risks and benefits. If your research is approved for a specific number of subjects, the data from any "extra" subjects cannot be described as having been obtained with IRB approval.

See the HSD website for the definition of "human subject" http://www.washington.edu/research/hsd/docs/1253. Before answering the questions below, be sure that you are familiar with the definition.

1. **Subject groups/categories and numbers.** Complete this table by listing:

- Your groups or categories of subjects. "Group" should be defined as appropriate for your research.
  - "Units" within a group. For most research, a group will consist of individuals, such as children aged 8-12, or individuals with high blood pressure. However, this will not be true for all research. Examples of groups with "units" that are not individuals:
    - Dyads such as Alzheimer's-patient-and-caregiver, with one group of the dyads assigned to one intervention (e.g., behavioral modification) and another group of the dyads assigned to a comparison intervention(e.g., drug treatment).
    - Families. For example, a study of mental health interventions for homeless families might have one group of 30 families assigned to one intervention and another group of 30 families assigned to a different intervention.
    - Other. For example, the "units" in autism research might be an autistic individual and all his/her living blood relatives. The units in an academic excellence study might be a student-parents-teacher unit.
  - Types of groups. There are many ways in which subjects might be grouped. Examples:
    - By intervention. Example: research comparing two different drugs for high blood pressure.
    - By subject population. Example: research comparing the incidence of domestic violence in families living in urban settings versus families living in rural settings.
    - If you have only one group, fill in only one line in the table. Add more lines if needed.
- The age range of each group.
- The upper limit/number of **completed** subjects you need for each group. ***Completed** means that all research procedures involving the subjects or the obtaining of specimens/records/data have been completed as far as is possible for each subject, including any follow-up (such as follow-up access to medical records.) In some cases, such as an online survey, it is not possible to predict the number of subjects who will complete the research. If you cannot predict or describe the maximum number of subjects you need in each group, check the appropriate box and provide your rationale in the space provided below the table.*

**Group name/description**	**Age range of subjects**	**Maximum desired number of individuals (or other group unit, such as families) who will complete the research.***	**Cannot provide a number.****
People with lower limb loss	>18	24	☐**
			☐**
			☐**
			☐**

***This is the number of subjects (individuals, dyads, families, etc., as appropriate) in each group that will be considered for approval by the IRB.**

****If you cannot predict or describe the maximum number of subjects you need in each group:**

Provide your rationale and description of research scope here. *Include any information or estimates you might have about the number of subjects, so that the IRB has a sense of the scope of your research. For example, your research might be a small pilot study of all patients presenting with a rare disease at UW Medicine in the next year. Or, it might involve a survey posted on Craig's List for two weeks that could result in thousands of responses.*

N/A

NOTE: In your periodic Status Report, you will be asked to complete the table below with your subject numbers. While developing your research protocol, please plan ahead so that you will have an accurate record of the subject numbers above.

This is for illustration only. Do not complete this table.

	# Completions				# Ongoing (subjects still involved)	# Withdrawals, drops, lost		
Group Name / Description	Total approved by IRB	A At time of last Status Report	B Since last Status Report	A + B Total to date		C At time of last Status Report	D Since last Status Report	C + D Total to date

DO NOT COMPLETE

2. Explain how you will achieve equitable subject representation in the following categories. If not applicable, justify exclusions.

   a. Age (minors, elderly):

   We will not be recruiting minor participants for this pilot study. At present, the mRSF is not regularly provided to children, so we will not be recruiting participants under the age of 18 for this study.

   b. Gender:

   We will not exclude participation based on gender. Both males and females are welcome to participate in this study.

   c. Ethnic and racial minority populations:

   We will not exclude participation based on ethnicity or race.

3. What characteristics (inclusion criteria) must subjects have to be in this study? (Answer for each subject group, if different.)

   Inclusion criteria include: 18 or more years of age, non-dysvascular unilateral transtibial amputation, prosthetic user for >1 year, are scheduled to receive a prosthesis with a mRSF, able to walk continuously for at least 6 minutes with or without an assistive device, able to read and write English.

4. What characteristics (exclusion criteria) would exclude subjects who are otherwise eligible from this study? (Answer for each subject group, if different.)

   Exclusion criteria include any health condition that would limit use of a prosthesis (e.g., skin breakdown), ability to safely walk for at least 6 minutes (e.g., heart disease), or participation (current or planned over the study period) in another research study that may affect the fit or function of his/her prosthesis.

5. Describe the subject recruitment strategies you will use for each group of subjects. (You should obtain letters of cooperation from agencies, institutions, or others involved in subject recruitment for your research records. Do not send these to HSD or the IRB.)

   Study participants will be exclusively recruited from Davidson Prosthetics, the only local prosthetics facility that routinely provides prostheses with ESF and mRSF to people with LLA. Informed consent will be obtained from all participants.

6. Explain who will approach subjects to take part in the study and how this will be done to protect subjects' privacy. (Attach letters of cooperation from agencies, institutions or others involved in subject recruitment.)

   Flyers will be posted at Davidson Prosthetics with study information and the phone number of the study coordinator (**Appendix 4)**. In addition, Mr. Davidson will let potential participants know about the study and provide the phone number of our study coordinator. Potential participants can call our study coordinator to get more information about the study, decide if they are interested in participating, and schedule their first study session (**Appendix 5**).

7. Explain what steps you will take during the recruitment process to minimize potential coercion or the appearance of coercion.

Their prosthetic care provider (Mr. Davidson) will only inform potential participants about the study. All recruitment and enrollment procedures will be conducted by study investigators/coordinators. All interested participants will be informed that participation (or not) is completely voluntary and will in no way affect the clinical care they receive. No members of our study team are or will be involved in the clinical care of potential participants or participants.

8. Will you give subjects gifts, payments, services without charge, or extra course credit? ☐ No ☒ Yes If yes, explain:

Subjects will receive $30 for every hour of participation in the screening session (Session 1). Participants will receive $50 for every hour of participant in data collection sessions (Sessions 2 & 3). Participants will be paid after each session.

9. Will any of the subjects or their third-party payers be charged for any study procedures? ☒ No ☐ Yes If yes, explain:

N/A

10. **UW Locations and research sites.** Provide the following information in list or table format for all UW locations at which any research procedures will occur. Be sure to consider: screening, recruiting, consenting, observation, intervention, data collection, data analysis, specimen analysis, and location of any consultants and collaborators.

- Geographical location and/or address
- Name of organization, agency, group, site, institution
- What procedures will occur at each location (how the location is involved in the research)
- Whether subject contact or interaction will occur at each site
- Whether consenting of subjects will occur at each site
- Whether each site, or individuals at the site, will obtain, use, or have access to coded or individually identifiable private information about subjects for research purposes

	Site 1:<br>University of Washington Department of Rehabilitation Medicine<br>Health Sciences Building, BB tower, 8th floor	Site 2:<br>Davidson Prosthetics, LLC<br>812 39th Avenue SW Suite D<br>Puyallup, WA 98373
Screening	X (Screening will be done by study investigators/staff over telephone)	
Recruiting	X (The study will be described to potential participants by study investigators/staff. Potential participants will have the opportunity to ask questions and decide if they would like to schedule a data collection session at this time)	X (Mr. Davidson will post flyers and let potential participants know about the study)
Consenting	X (Study investigators, Dr. Hafner and Ms. McDonald, will obtain informed consent at the data collection appointment, prior to data collection activities)	N/A
Observation	N/A	N/A
Intervention	N/A	N/A
Data Collection	X (Study investigators, Dr. Hafner and Ms. McDonald, will perform all data collection activities)	N/A
Data Analysis	X (Study investigators, Dr. Hafner and Ms. McDonald, will	N/A

	perform all data analysis activities)	

E. RISKS AND BENEFITS

In order to approve the research the IRB must find that risks are reasonable in relation to anticipated benefits, if any, to subjects, and the importance of the knowledge that may reasonably be expected to result.

1. Describe nature and degree of risk of possible injury, stress, discomfort, invasion of privacy, and other side effects from all study procedures, drugs and devices (standard and experimental), interviews and questionnaires. Include psycho-social risks as well as physiological risks. Include risks of withholding standard care or procedures if this is the case. Do not reference the consent form.

   Participants will be asked to walk for about 70 minutes total for this study (a maximum of 6 minutes at a time, and 30 minutes maximum per session). Because they are being asked to walk, there is the risk that they could become fatigued during the study procedures. In addition, there is a risk of falling. However, these risks are no larger than the risks that study participants generally undertake when walking outside of this study.

2. Explain what steps you will take to minimize risks of harm and to protect subjects' rights and welfare. (If you will include protected groups of subjects (minors; fetuses in utero; prisoners; pregnant women; unviable neonates; neonates of uncertain viability; decisionally impaired or economically or educationally disadvantaged subjects) please identify the group(s) and answer this question for each group. Please also complete the SUPPLEMENT: Protected and/or Vulnerable Populations.)

   This study will not require participants to perform activities at a level of endurance that they would not typically do outside of the study session. To minimize risks of fatigue and falling during the study session, the participants will be asked to take breaks after each walking measure is collected. The maximum time that each participant will be asked to walk continuously is 6 minutes. They are also instructed to take a break, including a sitting break, at any time during the data collection session, including in the middle of walking measures. If at any time it appears that the participant is at risk of falling or other harm, the data collection session will be stopped.

3. Is it possible that you will discover a subject's previously unknown condition (disease, suicidal intentions, genetic predisposition, etc.) as a result of study procedures? ☐ No ☐ Yes If yes, explain how you will handle this situation.

4. Describe the anticipated benefits of this research for individual subjects in each subject group. If none, state "None."

   None

5. Describe the anticipated benefits of this research for society.

   The mRSF is a promising advancement in prosthetic technology. However, currently, Mr. Davidson is one of few prosthetists in the United States that is providing this foot. Empirical evidence that supports its provision for people with LLA will help the mRSF become available to others who may benefit from its use.

F. ADVERSE EVENTS OR EFFECTS

1. Who will handle adverse events? ☒ Investigator ☐ Referral ☐ Other, explain:

   N/A

2. Are your facilities and equipment adequate to handle possible adverse events? ☒Yes ☐ No, explain:

   A first aid kit will be available, if needed, to address minor injuries (e.g., scrapes or minor cuts). The investigators will contact emergency services (911) if a serious adverse event occurs. The UW Medical Center emergency room is located 5 floors below the researchers' laboratory.

G. CONFIDENTIALITY OF RESEARCH DATA

1. Will you record any direct subject identifiers (names, Social Security numbers, patient, hospital, laboratory or claim numbers, addresses, telephone numbers, locator information, etc.) ☐ No ☒ Yes If yes, explain why this is necessary and describe the coding system you will use to protect against disclosure.

Subject identifiers will be recorded (name, telephone number, social security number). Name and contact information are needed to schedule participant data collection sessions. Social security information is required by UW when payments of more than $50 are made to study subjects. Identifying information will be linked to a 5-digit numeric study code. The file containing identifying information and the linked subject code will be stored separately from all other study data in a secure and password protected computer only accessible to study researchers.

2. Will you retain a link between study code numbers and direct identifiers after the data collection is complete? ☐ No ☒ Yes If yes, explain why this is necessary and for how long you will keep this link.

   This research is a pilot study in a new line of investigation for our research lab (i.e., same PI and research team). We are currently pursuing funding for parallel studies of prosthetic foot technologies. We wish to combine data from related studies to maximize our ability to recruit from the small local population of people with lower limb loss. Combining data from multiple studies would increase our power to detect differences and avoid participation of individuals in both studies (participation in both studies would lead to including a participant's data twice in the same dataset). To allow for accurate longitudinal tracking of participants, we will retain the link between identifiers and study code numbers until 2026. Participants may request that we destroy their link at any point during the study, or any time thereafter.

3. Describe how you will protect data against disclosure to the public or to other researchers or non-researchers. Explain who (other than members of the research team) will have access to data (e.g., sponsors, advisers, government agencies, etc.).

   Data will be stored on a study specific password protected computer which will be stored in a locked cabinet in the PI's office. Only members of the research team will have access to the information.

4. Will you place a copy of the consent form or other study information in the subject's medical or other personal record? ☒ No ☐ Yes. If yes, explain why this is necessary.

   N/A

5. Do you anticipate using any data (information, specimens, etc.) from this study for other studies in the future? ☐ No ☒ Yes If "Yes," explain and include this information in the consent form.

   As noted above, this research is a pilot study in a new line of investigation for our research lab. We are currently pursuing funding for similar (parallel) studies of the prosthetic foot technologies included in this study. To maximize available resources and address larger research questions, we will combine data from this study with data obtained in subsequent studies. We have added this information to the consent form.

H. ADDITIONAL INFORMATION

1. If the study will involve radiation exposure to subjects, e.g., X-rays, radioisotopes, what is status of review by the UW Radiation Safety Committee (RSC): ☐ Pending ☐ Approved (Attach one copy of approval.) ☒ NA

2. Does this research require approval from the UW Institutional Biosafety Committee (IBC) for recombinant/synthetic DNA human Gene transfer or vaccines?

   ☒ No ☐ Yes. If yes, what is the status of review by IBC? ☐ Pending ☐ Approved (Attach one copy of approval.) ☐ NA

3. <u>Protected Health Information (PHI)</u>. Will you or any member of your research team obtain, access, or use a subject's protected health information by any method, and for any purpose including "pre-screening"?

   "Methods" may include but are not limited to: directly looking at a medical record (electronic or paper), requesting medical record information from a service such as the UW Center for Health Excellence, or viewing surgery schedules, clinic records, appointment books, etc.

   Examples of where PHI may be located include: medical records, dental records, clinical lab tests that you will have performed on subject samples, pharmacy records, medical billing records, clinical databases, etc.

   ☐ No ☒ Yes. If "yes":

   a. Describe the type of records/data, location and how you will obtain the information:

We will collect information on the participants' prostheses, including delivery schedule, components, and problems experienced by the participants (related to fit and function of their prostheses) while they are part of the study. Information will be collected directly from the participant's practitioner (Mr. Davidson). Participants will be asked to complete a HIPAA authorization to allow us to collection information about their prosthesis directly from their practitioner (**Appendix 6**).

b. Will you obtain any of the information without HIPAA authorization from each subject?

☒ No ☐ Yes. If "yes": Complete and attach the SUPPLEMENT: Waiver Request, HIPAA Authorization, and the SUPPLEMENT: Waiver Request, Consent Requirements. If the records are owned by the University of Washington or a state agency, complete and attach a UW Confidentiality Agreement.

c. Will you obtain HIPAA authorization from subjects for any of the information?

☐ No ☒ Yes. If "yes", attach the HIPAA Authorization form you propose to use

See attachment (**Appendix 6**).

d. Will you be obtaining any of the data as a Limited Data Set?

☒ No ☐ Yes

4. Other Records. Will you or any member of your research team obtain, access, or use academic, employment, or any other type of records about subjects, by any method, and for any purpose including "pre-screening"?

"Methods" may include but are not limited to: directly looking at a record (electronic or paper), requesting records from offices such as Payroll or the UW Registrar's Office, obtaining records from the state Department of Health, etc.

☒ No ☐ Yes. If "yes":

a. Describe the type of records/data, location, and how you will obtain the information.

N/A

b. Will you obtain any of the information without the subject's consent?

☐ No ☐ Yes.

If the records are owned by the University of Washington, complete and attach a UW Confidentiality Agreement.

5. Will you use the Clinical Research Center (CRC) at the UW or Seattle Children's for any of your research activities?

☒ No ☐ Yes.

If you answered "yes":

A medical record will be created for your subjects at UW Medicine and CRC staff may need to access those medical records for you. This may be because they are performing procedures or collecting data for you. It may also be required if an event happens on the CRC that requires treatment (such as fainting during a blood draw). This means that you must obtain a signed HIPAA Authorization form from each subject and give a copy of it to the CRC. ***Complete and attach the UW research HIPAA Authorization template, available on the HSD Forms webpage. There is guidance in the template about how to describe the information that the CRC staff may access and disclose to you.***

6. Does your research involve any of the following:

- Students age 21 or younger who may be participants in your research?
- Access to, or use of, personally identifiable information from student (current or past) education records from any institution or agency of education (including, but not limited to, pre-elementary, secondary, post-secondary, job training, adult education, career and technical education, special education)?
- Conducting any research procedures in an educational setting?

☒ No ☐ Yes.

If you answered "yes":

Your research may be subject to the requirements of the **Protection of Pupil Rights Amendment (PPRA)** and/or the **Family Education Rights and Privacy Act (FERPA).**

Consult with the SOP Research Involving Students to determine whether PPRA or FERPA regulations apply to your research.

Check the appropriate box.

☐ PPRA regulations apply to my research

☐ FERPA regulations apply to my research

☐ Both PPRA and FERPA regulations apply to my research

☐ Neither set of regulations apply to my research

7. Will you make audio-visual or tape recordings or photographs of subjects? ☐ No ☒ Yes. If yes, explain what type of recordings you will make, how long you will keep them, and if anyone other than the members of the research team will be able to see them.

   With participant's consent, we will take video and/or photographs of study procedures for use in grant applications, presentations, or publications (**Appendix 7**). Consent for photos/videos is not required to participate in the study. We will not destroy photos and/or videos.

8. Will your study involve use of equipment involving energy input to the subjects (EMG, EKG, MRI, ultrasound, etc.)? ☒ No ☐ Yes. If yes, attach documentation that all equipment will be tested regularly by the Scientific Instrument Division (call (206) 543-5580 for information) or describe safety testing procedures you will use.

   N/A

9. Confirm by checking the box that the principal investigator on this IRB application has ensured that all investigators (as defined by UW policy GIM 10) are aware of policy GIM 10 and their responsibility for complying with its relevant requirements.

   ☒ Confirmed

10. Does the individual who is the principal investigator on (1) this IRB application or (2) any grants or contracts supporting this research have a financial conflict of interest with respect to this research? ☒ No ☐ Yes.

    If yes, has it been disclosed to the University? (Since August 24, 2012, all disclosures are made through the University's online Financial Interest Disclosure System.) Final review of this application cannot occur until the disclosure has been made and reviewed by the University, and the outcome has been incorporated into the IRB's review. ☐ No ☐ Yes ☐ Not applicable, because there is no financial conflict of interest.

11. Is your research:

    - Clinical research that will bill subjects or their health insurance for UW Medicine professional or facility services, items, or tests*, **AND/OR**
    - An "applicable clinical trial" as defined below" **

      ☐ No ☒ Yes

    If you selected "yes", you must register your research at the federal site ClinicalTrials.gov

    See the HSD document titled: ClinicalTrials.gov – Instructions for Registering Your Trials for step-by-step instructions about how to register your research.

    ***New Requirement**

    As of January 1, 2014, a new federal requirement will require you to provide the clinical trials registration number assigned to your research in order to bill most UW medicine professional or facility services, items, or tests to research participants or their health insurance. This new billing requirement applies to some clinical research, such as Phase I studies, that don't meet the federal registration definition of "applicable clinical trials". *See also*: Clinical Research Budget & Billing Support (CRBB)

    ***Applicable clinical trial is defined as:*

    (1) a pediatric postmarket surveillance study required by the FDA ***OR***

    (2) an interventional study (with one or more arm) of an FDA-regulated drug, biological product, or device that involves health outcomes and meets one or more of the following conditions:

    - *The trial has at least one site in the United states; or*
    - *The trial is conducted under an FDA investigational new drug application or investigational device exemption; or*

- *The trial involves a drug, biologic, or device that is manufactured in the United States or its territories and is exported for research*

The source of funding (e.g., industry, federal, nonprofit) is irrelevant.

See this website for additional information: http://prsinfo.clinicaltrials.gov/ElaborationsOnDefinitions.pdf

I. CONSENT

Obtaining informed consent is a process that involves more than obtaining a signature on a form. It is a process of information exchange that may include subject recruitment materials, verbal instructions, question-and-answer sessions, and measures of participant understanding. Obtaining voluntary informed consent is one of the central protections required by all human subjects regulations and ethical principles. The key features of the consent process include:

- Disclosure of the information needed to make an informed decision about participation
- Facilitation of comprehension by the potential participant
- Promotion of the voluntariness of the potential participant's decision

Refer to the **SOP Consent** and **SOP Consent Documentation** for more information.

1. Description of consent process for adult subjects. How are you going to obtain informed consent from your adult subjects? Describe in detail your consent methods, process, and settings. Identify who will provide the information to subjects and who will interact with them during the consent process. If there is more than one consent process, describe each one separately. For subjects who do not speak English: Describe the process that will be used, and whether anyone on the research team will speak the subjects' language. **Complete this section if you will obtain consent from any subjects for any aspect of the research.**

   Potential participants will provide informed consent at their scheduled study session, but before any data collection procedures. A study investigator will describe the study to the participant using the written consent form (**Appendix 1**) as guide, allow time for the potential participant to read through the consent form, ask if the participants have any questions, and ask them to sign the form if they are interested in participating. If the potential participant is not interested, they will not be asked to perform any study procedures.

2. Description of assent process for children subjects. Describe in detail how you will obtain assent from children subjects, following the instructions provided in the question above. Also, describe how these processes will differ based on age/cognitive ability. Finally, describe how you will determine whether a child is assenting or dissenting throughout the research (if applicable). **Assent means a child's affirmative agreement to participate in the research. Mere failure to object should not be interpreted as assent.*

   N/A

3. Special issues or considerations. The standard concept of consent is based on the Western ethical tradition of individual autonomy and privacy. This may not apply well to your research. Your research may be subject to specific cultural or other contextual issues that affect the consent process. Describe any special issues and considerations about obtaining consent for your research. If none, state: "Not Applicable".

   Example issues:

   - Who is the appropriate person(s) for providing consent?
   - The desirability of a group consent process, or a surrogate consent process
   - Research that occurs in a setting with a blurred sense of what is public versus private
   - The cultural acceptability of the consent process (or documentation)
   - Cultures or groups in which it is considered impolite to refuse a request and/or in which people are fearful of refusing requests that they regard as coming from authorities

   N/A

4. Undue influence. Describe how you will minimize any undue influence on your subjects' decision about participating in your research. If this is not an issue for your research, describe why. *This is an important consideration when persons recruiting or consenting subjects are in a position of authority or influence – for example, the subject's teacher, doctor, or employer.*

   Potential participants will be identified via flyers (**Appendix 4**) posted in their prosthetic office. However, all other recruitment and consenting procedures will be

done by study investigators who are not involved in potential participant's health care. Study investigators will stress that participation is voluntary and in no way will participation (or non-participation) in the study affect their prosthetic (or other health) care.

5. Subject comprehension. Describe anything that you will do to facilitate or verify your subjects' comprehension of the information you provide them during the consent process.

Potential participants will be encouraged to ask questions throughout the consent process.

6. Do you expect that all of your participants will be **fluent** in spoken and/or written English? ☐ No ☒ Yes.

If "No", please answer the following questions.

6.1. In what language(s) will they be fluent?

N/A

6.2. Translation of documents into another language. Federal regulations require that consent, assent, and authorization documents must be presented to participants in a language that is understandable to them. The UW IRB expects that translated documents will be:

- Linguistically accurate;
- At an appropriate reading level for the subject population; and
- Culturally sensitive for the locale in which they will be used.

Describe how you will obtain translations of relevant documents, and how you will ensure that the translations meet these requirements.

N/A

6.3. Interpretation. Describe how you will provide interpretation, and when. Specifically:

a. For what situations will you provide interpretation? (At a minimum, an interpreter should be available for the consent process, unless the IRB has waived consent.)

N/A

b. Who will be the interpreter?

N/A

c. Describe the qualifications of the interpreter – for example, background, experience, language proficiency in English and in the other language, native language fluency, certification, other credentials, familiarity with the research-related vocabulary in English and the target language.

N/A

d. How will you ensure that the subjects will understand ongoing study-related communication? If the subject has questions, complaints, or adverse events, how will that be communicated to the researchers?

N/A

7. Check all that apply:

☒ **Written** *Attach copies of all consent forms for each subject group. Include a footer identifying the version date of each form and a header or title that identifies each different form. If you propose to delete one or more of the required elements of consent from a consent form, attach and complete the form called SUPPLEMENT: Waiver Request, Consent Requirements.*

See attachment (**Appendix 1**).

☒ **Waiver of written documentation of consent** *This means that you are requesting a waiver of the requirement to obtain written documentation of consent. Complete and attach the form called SUPPLEMENT: Waiver Request, Consent Requirements. . Also, attach the Information Statement, oral consent or assent protocol and script, or other materials you will use to communicate the necessary elements of consent to the subjects.*

See attachment (**Appendix 8**).

☐ **Waiver of consent** *This means that you are requesting a waiver of the requirement to obtain consent. Complete and attach the form called SUPPLEMENT: Waiver Request, Consent Requirements. .*

☐ **Assent** *Attach copies of any written materials or scripts you will use with minor subjects (individuals under the age of 18) to obtain their assent to being in your research.*

☐ **Parental permission** *Attach copies of any written materials or scripts you will use with parents, to obtain their permission to enroll their minor children in your research. See also SUPPLEMENT: Protected and/or Vulnerable Populations for waivers or alterations of consent requirements.*

UNIVERSITY of WASHINGTON
HUMAN SUBJECTS DIVISION
Box 359470
Seattle, WA 98195-9470
Phone: 206-543-0098
Fax: 206-543-9218

SUPPLEMENT: Devices

Click to reveal all branching
(on first device form only)

1. Research Study Information

FOR HSD OFFICE USE ONLY
DATE RECEIVED STAMP:

Lead Researcher Name:

Brian J. Hafner, PhD

Full Application Title:

Evaluation of a Modified Running Prosthesis

IRB Application Number (if known):

IRB Committee (if known):

This form is being submitted as an attachment to:

- (●) IRB Initial application
- ( ) IRB Modification application
- ( ) Other (describe):

END PART ONE

Number of devices included on this form: 1
(Calculates as devices are added using the button at the end of the form.)

Device Description

Answer the following questions for each device

Device #: 1

Basic Descriptive Information

1. Describe the device. Include: generic or common name; brand name (if applicable); model number (if applicable); purpose; function/operations.

Participants will be tested while wearing custom fabricated prostheses that have been prescribed, fitted, and delivered directly to them by their medical providers. These prostheses include prosthetic feet which are classified by the FDA as Class 1 devices and are exempt (510(k)) from the GMP regulation. Example photos and the FDA Exemption Notification are attached to this form.

Attach a copy of any informational documentation about the item, including as many of the following as possible (if applicable): device manual or similar documentation; package insert; photo or drawing; etc.

2. Is this a device that will be implanted in the participants?

- ( ) **YES**
- (●) **NO**

3. Who is providing you with the device for this research use?

- ( ) Commercial manufacturer (such as a device company)
- ( ) A non-commercial manufacturer (such as a research lab - your own or someone else's) or a non-profit that is making it (e.g. PATH).
- (●) Other

Describe who, where, how, and when:

The devices will be fabricated, fit, and provided by the participants' clinical provider (Greg Davidson, CPO), a licensed and certified prosthetist.

Use of the Device

4. Select the most appropriate description of the FDA Status the device as it is being used.

(●) FDA-approved device that is not a Humanitarian Use Device that is being used "on-label" (i.e. FDA-approved purpose, population, manner) EXEMPT

You are now done with this form. The remainder of the form, except for question 10, has been hidden. Go to question 10 to add another device. If you wish to change your selection for the use of the device, deselect the "FDA-approved" option and the remainder of the form will reappear.

Add Device

10. Are you using another device?

( ) **YES**

(●) **NO** You are done with this form.

Examples of Prostheses with different types of Prosthetic Feet. Prosthetic Feet are exempt from FDA GMP regulation (FDA Regulation Number 890.3420)

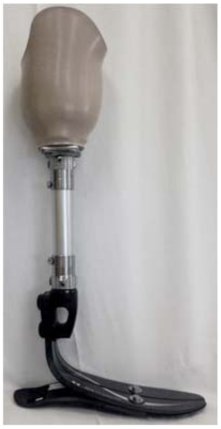

Prosthesis with Energy Storing Foot (ESF)

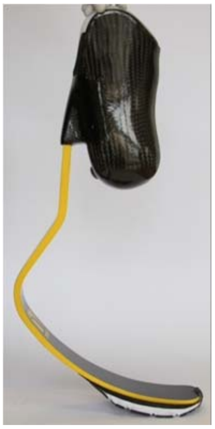

Prosthesis with Running Specific Foot (RSF)

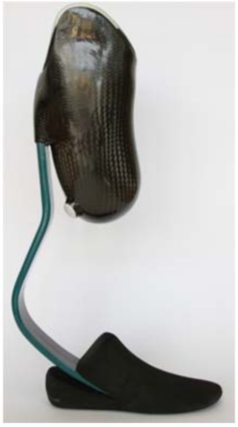

Prosthesis with Modified RSF (mRSF)

Product Classification

New Search Back To Search Results

**Device**	Component, External, Limb, Ankle/Foot
**Regulation Description**	External limb prosthetic component.
**Regulation Medical Specialty**	Physical Medicine
**Review Panel**	Physical Medicine
**Product Code**	ISH
**Premarket Review**	Office of Device Evaluation[6](ODE)<br>Division of Neurological and Physical Medicine Devices (DNPMD)<br>Physical Medicine and Neurotherapeutic Devices Branch (PNDB)
**Submission Type**	510(K) Exempt
**Regulation Number**	890.3420[7]
**Device Class**	1
**Total Product Life Cycle (TPLC)**	TPLC Product Code Report[8]
**GMP Exempt?**	Yes

Note: This device is also exempted from the GMP regulation, except for general requirements concerning records (820.180) and complaint files (820.198), *as long as the device is not labeled or otherwise represented as sterile.*

Note: FDA has exempted almost all class I devices (with the exception of reserved devices[9]) from the premarket notification requirement, including those devices that were exempted by final regulation published in the *Federal Registers* of December 7, 1994, and January 16, 1996. It is important to confirm the exempt status and any limitations that apply with 21 CFR Parts 862-892[10]. Limitations of device exemptions are covered under 21 CFR XXX.9, where XXX refers to Parts 862-892.

If a manufacturer's device falls into a generic category of exempted class I devices as defined in 21 CFR Parts 862-892[11], a premarket notification application and fda clearance is not required before marketing the device in the U.S. however, these manufacturers are required to register their establishment. Please see the Device Registration and Listing website[12] for additional information.

**Third Party Review**	Not Third Party Eligible

Links on this page:

1. http://www.addthis.com/bookmark.php?u508=true&v=152&username=fdamain
2. http://www.addthis.com/bookmark.php
3. http://www.fda.gov/default.htm
4. http://www.fda.gov/MedicalDevices/default.htm
5. http://www.fda.gov/MedicalDevices/DeviceRegulationandGuidance/Databases/default.htm
6. http://www.fda.gov/AboutFDA/CentersOffices/OfficeofMedicalProductsandTobacco/CDRH/CDRHOffices/ucm127854.htm#ODE
7. ../cfcfr/cfrsearch.cfm?fr=890.3420
8. ../cfTPLC/tplc.cfm?id=4748
9. ../cfpcd/3151.cfm
10. ../cfcfr/cfrsearch.cfm?cfrpartfrom=862&cfrpartto=892
11. ../cfcfr/cfrsearch.cfm?cfrpartfrom=862&cfrpartto=892
12. http://www.fda.gov/medicaldevices/deviceregulationandguidance/howtomarketyourdevice/registrationandlisting/default.htm

Page Last Updated: 12/15/2014

Note: If you need help accessing information in different file formats, see Instructions for Downloading Viewers and Players.

Accessibility Contact FDA Careers FDA Basics FOIA No Fear Act Site Map Transparency Website Policies

U.S. Food and Drug Administration
10903 New Hampshire Avenue
Silver Spring, MD 20993
Ph. 1-888-INFO-FDA (1-888-463-6332)
Email FDA

For Government For Press

Combination Products Advisory Committees Science & Research Regulatory Information Safety Emergency Preparedness International Programs News & Events Training and Continuing Education Inspections/Compliance State & Local Officials Consumers Industry Health Professionals FDA Archive

U.S. Department of Health & Human Services

Links on this page:

FDA Home[3] Medical Devices[4] Databases[5]

Establishment Registration & Device Listing

New Search Back To Search Results

**Proprietary Name:**	Adult Adapters; Aspire; Aspire BK Locking Components; Aspire Foot; Balance Vacuum; Cheetah Xtend; Cheetah Xtreme; Cheetah® Junior; Cheetah® Xplore Junior; Chopart; D/P Flexion; Elation; Fabrication Kits for Locks; Fixed Offset Adapter; Flex-Foot Assure; Flex-Foot Axia; Flex-Foot Balance; Flex-Foot Junior; Flex-Run; Flex-Symes; Foot Covers & FF Socks; Icelock 100; Icelock 200; Icelock 214 Offset AK; Icelock 300; Icelock 400; Icelock 500; Icelock 600; Icelock 700; Icelock Adapters; Iceross Activa; Iceross Casting Liners; Iceross Comfort Cushion; Iceross Comfort Locking; Iceross Dermo Conical; Iceross Dermo Cushion; Iceross Dermo Locking; Iceross Dermo Seal-In; Iceross Junior; Iceross Original; Iceross Seal-In V (TT); Iceross Seal-In X TF; Iceross Seal-In X5 (TF); Iceross Seal-In X5 (TT); Iceross Special Order; Iceross Sport; Iceross Stabilo Seal-In; Iceross Synergy; Iceross Synergy Cushion; Iceross TF Seal-In (HSM); Iceross Transfemoral; Iceross Upper X; Junior Adapters; K2 Sensation; LP Vari-Flex & Foot Adapter; Modular III; Nike Sole; Ossur 100 Foot; Proprio Foot; Re-Flex Rotate; Re-Flex Shock; Re-Flex VSP & Ceterus; Shock Absorbers; Socks and Sheaths; Spare Parts for Locks; Sport Feet; Sure-Flex; Talux; TF Unity Accessory kit; THE PIN-ICELOCK 400; Vari-Flex; Vari-Flex Modular; Vari-Flex XC; Vari-Flex XC Rotate; Vari-Flex® Junior
**Classification Name:**	COMPONENT, EXTERNAL, LIMB, ANKLE/FOOT
**Product Code:**	ISH[6]
**Device Class:**	1
**Regulation Number:**	890.3420[7]
**Medical Specialty:**	Physical Medicine
**Registered Establishment Name:**	OSSUR H/F[8]
**Registered Establishment Number:**	3003764610
**Owner/Operator:**	OSSUR H/F[9]
**Owner/Operator Number:**	9026971
**Establishment Operations:**	Manufacturer; Specification Developer

---

Links on this page:

1. http://www.addthis.com/bookmark.php?u508=true&v=152&username=fdamain
2. http://www.addthis.com/bookmark.php
3. http://www.fda.gov/default.htm
4. http://www.fda.gov/MedicalDevices/default.htm
5. http://www.fda.gov/MedicalDevices/DeviceRegulationandGuidance/Databases/default.htm
6. ../cfPCD/classification.cfm?ID=4748

7. ../cfCFR/CFRsearch.cfm?FR=890.3420
8. ../cfRL/rl.cfm?rid=16530
9. ../cfRL/rl.cfm?start_search=1&OwnerOperatorNumber=9026971

Page Last Updated: 12/15/2014

Note: If you need help accessing information in different file formats, see Instructions for Downloading Viewers and Players.

Accessibility Contact FDA Careers FDA Basics FOIA No Fear Act Site Map Transparency Website Policies

U.S. Food and Drug Administration
10903 New Hampshire Avenue
Silver Spring, MD 20993
Ph. 1-888-INFO-FDA (1-888-463-6332)
Email FDA

For Government For Press

Combination Products Advisory Committees Science & Research Regulatory Information Safety Emergency Preparedness International Programs News & Events Training and Continuing Education Inspections/Compliance State & Local Officials Consumers Industry Health Professionals FDA Archive

U.S. Department of **Health & Human Services**

Links on this page:

CFR - Code of Federal Regulations Title 21

[Code of Federal Regulations]
[Title 21, Volume 8]
[Revised as of April 1, 2014]
[CITE: 21CFR890.3420]

TITLE 21--FOOD AND DRUGS
CHAPTER I--FOOD AND DRUG ADMINISTRATION
DEPARTMENT OF HEALTH AND HUMAN SERVICES
SUBCHAPTER H--MEDICAL DEVICES

PART 890 -- PHYSICAL MEDICINE DEVICES

Subpart D--Physical Medicine Prosthetic Devices

Sec. 890.3420 External limb prosthetic component.

(a) *Identification.* An external limb prosthetic component is a device intended for medical purposes that, when put together with other appropriate components, constitutes a total prosthesis. Examples of external limb prosthetic components include the following: Ankle, foot, hip, knee, and socket components; mechanical or powered hand, hook, wrist unit, elbow joint, and shoulder joint components; and cable and prosthesis suction valves.

(b) *Classification.* Class I (general controls). The device is exempt from the premarket notification procedures in subpart E of part 807 of this chapter, subject to the limitations in 890.9. The device is also exempt from the current good manufacturing practice requirements of the quality system regulation in part 820 of this chapter, with the exception of 820.180, regarding general requirements concerning records and 820.198, regarding complaint files.

[48 FR 53047, Nov. 23, 1983, as amended at 66 FR 38816, July 25, 2001]

Links on this page:

1. http://www.addthis.com/bookmark.php?u508=true&v=152&username=fdamain
2. http://www.addthis.com/bookmark.php
3. http://www.fda.gov/default.htm
4. http://www.fda.gov/MedicalDevices/default.htm
5. http://www.fda.gov/MedicalDevices/DeviceRegulationandGuidance/Databases/default.htm
6. /scripts/cdrh/cfdocs/search/default.cfm?FAQ=true
7. http://www.fda.gov/MedicalDevices/DeviceRegulationandGuidance/Databases/ucm135680.htm
8. /scripts/cdrh/devicesatfda/index.cfm?Search_Term=External%20limb%20prosthetic%20component%2E

Page Last Updated: 09/01/2014

Note: If you need help accessing information in different file formats, see Instructions for Downloading Viewers and Players.

Accessibility Contact FDA Careers FDA Basics FOIA No Fear Act Site Map Transparency Website Policies

U.S. Food and Drug Administration
10903 New Hampshire Avenue
Silver Spring, MD 20993
Ph. 1-888-INFO-FDA (1-888-463-6332)
Email FDA

For Government For Press

Combination Products Advisory Committees Science & Research Regulatory Information Safety Emergency Preparedness International Programs News & Events Training and Continuing Education Inspections/Compliance State & Local Officials Consumers Industry Health Professionals FDA Archive

U.S. Department of **Health & Human Services**

Links on this page:

From: Shannon Sewards <ssewards@uw.edu>
To: "Geri C. Faris" <gfaris@uw.edu>, "Brian J. Hafner" <bhafner@uw.edu>
Subject: Re: FDA question
Date: Thu, 18 Dec 2014 23:41:48 +0000
On Dec 18, 2014, at 3:15 PM, Shannon Sewards <ssewards@uw.edu> wrote

Dear Dr. Hafner,

Your email was forwarded to me so that I may assist you with your request. From your email, it appears that you are using a device that has 510k approval from the FDA. If you will be using the device in your study according to its approved indication, there is no need for an IDE. Here is the regulatory reference for this determination:

21 CFR 812.2 c) *Exempted investigations.* This part, with the exception of 812.119, does not apply to investigations of the following categories of devices:

(1) A device, other than a transitional device, in commercial distribution immediately before May 28, 1976, when used or investigated in accordance with the indications in labeling in effect at that time.

(2) A device, other than a transitional device, introduced into commercial distribution on or after May 28, 1976, that FDA has determined to be substantially equivalent to a device in commercial distribution immediately before May 28, 1976, and that is used or investigated in accordance with the indications in the labeling FDA reviewed under subpart E of part 807 in determining substantial equivalence.

I hope this helps. If any additional information is needed, please do not hesitate to contact me.

Kind regards,

Shannon

Shannon Sewards, MA, CIP | Associate Director, Human Subjects Division, University of Washington
P: 206-543-2254 | F: 206-543-9218 | E: ssewards@uw.edu | Mail Box: 359470

UNIVERSITY OF WASHINGTON
Department of Rehabilitation Medicine

Evaluation of a Modified Running Prosthesis

Needed: People over the age of 18 years with unilateral non-dysvascular transtibial amputation who have worn a prosthesis for >1 year and are transitioning to a Modified Running-Specific Foot ("Modified Cheetah")

Topic: How a modified running specific prosthetic foot affects endurance, walking, mobility, and perceived exertion.

Purpose: To understand how the Modified Cheetah affects function

Time: 3 session at the University of Washington Medical Center, Seattle, WA
Total Time: 7.5 hours.

Protocol: Walking inside for up to 30 minutes, both on a treadmill and in a hallway. You will also be asked questions about yourself, your health, and your prosthesis.

Risks: Becoming tired, but you may stop to rest at any time. Falling while walking.

Participation in this project is voluntary. You will receive $50 per hour for participation in this study.

Contacts:
Cody McDonald
codym@uw.edu
206-221-6347

*Please note that we cannot guarantee the confidentiality of email communications.

<u>Evaluation of a Modified Running Prosthesis</u>

Telephone Screening Questionnaire

<u>POTENTIAL PARTICIPANTS MUST BE SCREENED, NO PROXY RESPONSES.</u>

Hello, this is [insert name] from the Modified Running Prosthesis study at the University of Washington. I am returning your call about the study. Is this a good time to talk? (*Or if the subject has called:* Thank you for calling about the study).

If you are interested, I would like to tell you about the purpose of this project, the risks and benefits of participating and the procedures involved. I will also need to ask you some questions to see if the study is a good fit for you. This should take about 10 minutes. Any information you give me will be kept in confidence, and you are free not to answer any questions you do not wish to answer. If it is determined that the study is not a good fit for you, the information from this screening interview will be destroyed. Are you interested in hearing more about the project?

Purpose: The purpose of the study is to evaluate walking in people who use different types of prosthetic feet. In this study, we will compare people's ability to walk in a typical "energy storing" foot to their ability to walk in a modified running foot called the "Modified Cheetah."

Location/time commitment: The information for this study will be collected during three visits that will last approximately 7.5 hours in total. All study sessions will be conducted at the University of Washington Medical Center.

Procedures: During Session 1, you will first be asked to complete a survey that includes medical and demographic questions. Medical questions include questions about the date and cause of your amputation. Demographic questions include questions about your age and education level. You can choose to leave any survey questions blank if you would prefer not to answer them. We will be attaching a random study identifier to your information to prevent a direct link between your personal information and the study data.

You will then be asked to walk on a treadmill in your current prosthesis to determine your comfortable, slow, and fast walking speeds. You will be prompted to adjust the treadmill until you determine your comfortable walking speed. This will then be recorded for use at later sessions. You will then follow this same process to establish your slow and fast speeds. Once your fast speed is selected, you will be asked to walk for 6 minutes at this speed to ensure that it isn't too fast to maintain for 6 minutes. You can slow down at any time.

After this 1st session, you will be assigned to one of two groups. Group 1 will wear the prosthesis with the Modified Cheetah first, then transition to the prosthesis with the energy storing foot. Group 2 will wear the energy storing foot first, then transition to the Modified Cheetah foot.

You will then schedule appropriate appointments with Greg Davidson to receive the selected prosthesis. We will then schedule a second session with him after you have received and worn your new prosthesis for at least 1 month.

During Session 2, you will be asked to complete the following study procedures with whichever prosthesis you were randomized to wear first.

- You will be asked to complete four electronic surveys about mobility, fatigue, balance confidence, activity restrictions, and satisfaction.
- You will then be asked to put on a small machine that measures your oxygen consumption. You will sit quietly for 5 min. so we can measure how much oxygen you use when you are resting. You will then stand quietly for 5 min. so we can measure your standing oxygen consumption. You will then remove the machine.
- You will then be asked to complete walking test called the Six-Minute Walk Test. For this test, you will be asked to walk back and forth between two cones for 6 minutes. You will perform this test on a walking mat that will record your steps. You may rest at any point and a chair will be brought to you if you would like to sit during the test. You will be instructed to walk as quickly as possible during this test. You may use an assistive device, such as a cane or walker, when performing this test. We will record the total distance you walked during the test.
- Following the Six-Minute Walk Test, we will ask you to report your level of exertion on a scale from 0-100. A rating of 0 indicates no exertion at all and a rating of 100 indicates maximal exertion. This test will take under one minute.
- You will then be asked to put the small machine back on, to wear during your three walking sessions on the treadmill. You will walk on the treadmill for 6 minutes per session for three sessions at slow, comfortable, and fast speeds in a randomized order. Between each session you will rest for at least 5 minutes.
- This session will take approximately 3 hours.

After this session, you will schedule an appointment to return to Davidson Prosthetics to switch to the other prosthesis and foot. After you have worn the second prosthesis for at least 1 month, you will schedule your third and final session with us. This 3rd session will be identical to session 2, but wearing a different prosthesis.

You may choose not to complete any of these tests at any time during the session.

You will receive $30 per hour for Session 1 and $50 per hour for Session 2 and 3, as well as parking reimbursement for visits to the University of Washington. You will need to provide your own transportation to the University of Washington Medical Center in Seattle, WA.

Risks of being a study participant: If you choose to participate in this study, you will be asked to walk for about 30 minutes during each session. Because you will be asked to walk for this length of time, there is a risk that they could become tired. In addition, there is a risk that you may fall while walking. To minimize risks of fatigue and falling please let study investigators know if you would like to take a break and/or sit down. You are welcome to take a break at any time during the session. If at any time it appears that you are at risk of falling or other harm, study investigators will end the data collection session.

Benefits of being a study participant: You will not directly benefit from taking part in this study. However, the Modified Cheetah is a promising prosthetic foot. Research involving this foot will help make it available to others who may benefit from its use.

Voluntary Nature of Participation: Participation in this study is strictly voluntary. You do not have to participate in this study. If you decide not to participate in this study, it will not affect any services you receive at the University of Washington Medical Center or at Davidson Prosthetics. If you do agree to participate and then decide to stop, you can withdraw at any time.

Do you have any questions about what I have described?

Eligibility for the study: Now I would like to ask you some questions to see if you meet the criteria for our study.

1) Are you 18 years of age or older?	**YES**	**no**
2) Do you have an amputation below your knee on one side of your body with no other amputations?	**YES**	**no**
3) Was the date of your amputation over one year ago?	**YES**	**no**
4) Was the cause of your amputation related to vascular problems?	**YES**	**no**
5) Are you in good health?	**YES**	**no**
6) Do you currently have any wounds or sores on your residual limb?	**YES**	**no**
7) Are you being fitted with a new prosthesis with a Modified Cheetah foot at Davidson Prosthetics in the next 3 months?	**YES**	**no**
8) Are you able to walk for at least 6 minutes without assistance from another individual or use of an assistive device?	**YES**	**no**
9) Are you currently participating or planning to participate in another prosthetic research study in the next three months?	**YES**	**no**
10) Are you able to read and write in English	**YES**	**no**

If the subject answers 'no' to any of the above, they do not qualify for the study:
"Thank you for your interest in this study. Our study is not a good fit for you at this time. I will destroy all the information related to this telephone screen. Thank you again for your time."

If the subject meets the initial eligibility criteria, they are eligible for the study: Thank you so much for your interest in our study and your patience with this telephone screen. We would like to include you in our study. Are you interested in volunteering? If so, I need your name and contact information, and then we can schedule an appointment at your convenience.

Name: ____

Address: ____

Phone: ____

(Schedule participant for appointment)

Appointment Date and Time: ____

Thank you for your time and assistance. We will be sending you a letter of confirmation with your appointment time and location and how to contact us if you should have further questions.

HIPAA Authorization

For the Use of Patient Health Information for Research

Research Title: Evaluation of a Modified Running Prosthesis
Lead researcher: Brian Hafner, PhD
Institution of lead researcher: University of Washington

A. Purpose of this form

The purpose of this form is to give your permission to the research team to obtain and use your patient health information. Your patient information will be used to do the research named above.

State and federal privacy laws protect your patient information. These laws say that, in most cases, your health care provider can release your identifiable patient information to the research team only if you give permission by signing this form.

You do not have to sign this permission form. If you do not, you will not be allowed to join the research study. Your decision to not sign this permission will not affect any other treatment, health care, enrollment in health plans or eligibility for benefits.

B. The patient information that will be obtained and used

"Patient information" means the health information in your medical or other healthcare records. It also includes information in your records that can identify you. For example, it can include your name, address, phone number, birthdate, and medical record number.

1. Location of patient information

By signing this form you are giving permission to the following organization(s) to disclose your patient information for this research.

Davidson Prosthetics, LLC____

____

2. Patient information that will be released for research use

This permission is for the health care provided to you during the following time period: from the time you received your most recent prosthesis until the end of this research study.

The specific information that will be released and used for this research is described below*:*

- Dates associated with fabrication and delivery of your prosthesis (e.g., fabrication dates, delivery dates)
- Details about your prostheses (e.g., components, alignment, adjustments)
- Issues you may experience with fit and function of your prosthesis (e.g., skin breakdown, socket discomfort)
- Modifications to your prosthesis made to address fit or function issues (e.g., pads, socket modifications)

C. How your patient information will be used

The researcher will use your patient information only in the ways that are described in the research consent form that you sign and as described here.

The research consent form describes who will have access to your information. It also describes how your information will be protected. You can ask questions about what the research team will do with your information and how they will protect it.

The privacy laws do not always require the receiver of your information to keep your information confidential. After your information has been given to others, there is a risk that it could be shared without your permission.

D. Expiration

This permission for the researchers to obtain your patient information:
ends on March 31, 2017.

E. Canceling your permission

You may change your mind at any time. To take back your permission, you must send your **written** request to:

Brian Hafner, PhD
University of Washington
1959 NE Pacific St
UW Box 354237
Seattle, WA 98195

If you take back your permission, the research team may still keep and use any patient information about you that they already have. But they can't obtain more health information about you for this research unless it is required by a federal agency that is monitoring the research.

If you take back your permission, you will need to leave the research study. Changing your mind will not affect any other treatment, payment, health care, enrollment in health plans or eligibility for benefits.

F. Giving permission

You give your permission to release your information by signing this form.

____
Printed Name of Research Subject Birthdate

____
Signature of Research Subject Date of signature

You will receive a copy of this signed form. Please keep it with your personal records.

UNIVERSITY OF WASHINGTON
PHOTOGRAPH & VIDEO RECORDING PUBLICATION CONSENT FORM

Evaluation of a Modified Running Prosthesis

Researchers: Brian Hafner, PhD, Associate Professor, Division of Prosthetics and Orthotics, Department of Rehabilitation Medicine, 206-685-4048

Patricia Kramer, PhD, Associate Professor, Department of Anthropology, 206-616-2449

Cody McDonald, L/CPO, Graduate Research Assistant, Division of Prosthetics and Orthotics, Department of Rehabilitation Medicine, 206-221-6347

Researchers' statement

USES OF THE PHOTOGRAPHS AND/OR VIDEO RECORDINGS

We are interested in knowing how walking changes when people walk with different types of prosthetic feet. We may want to use the photographs and the video recordings that we take during your session for educational or academic presentations or in academic reading material.

We would like to keep the following photos and video recordings to use for our research. It is possible for someone who knows you to recognize your face, body, or clothing.

We ask your permission to use the following photographs and video recordings in academic public presentations, academic written material, and in educational settings.

____

____

____

You have been given an opportunity to review the above photograph(s) and/or video recording(s) and we request your permission for the research team to use them in the following way:

- ☐ Academic public presentations
- ☐ Educational settings
- ☐ Academic journals
- ☐ Keep the photographs and/or recordings for research purposes indefinitely

____________________________ ______________________________ _______

Printed name of researcher Signature of researcher Date

Subject's statement

I have had an opportunity to review the photograph(s) and/or video recording(s) referenced above. I give my permission to the researchers to use the items as I have indicated above in this consent form. I understand that my name will not be published in connection with any such presentation or publication. I will not receive any compensation for the use of the recordings or photographs. I will receive a copy of this consent form.

____________________________ ______________________________ _______

Printed name of subject Signature of subject Date

UNIVERSITY of WASHINGTON
HUMAN SUBJECTS DIVISION
Box 359470
Seattle, WA 98195-9470
Phone: 206-543-0098
Fax: 206-543-9218

SUPPLEMENT:
Waiver Request, Consent or Consent Requirements

1. Research Study Information

PART 1

Lead Researcher Name:

Brian Hafner, PhD

Full Application Title:

Evaluation of a Modified Running Prosthesis

IRB Application Number (if known):	IRB Committee (if known):

This form is being submitted as an attachment to:

[X] IRB Initial application
[ ] IRB Modification application
[ ] Other.....describe:

FOR HSD OFFICE USE ONLY
DATE RECEIVED STAMP:

2. Nature of Request

Indicate which of the following you are requesting, and then complete the relevant sections

PART 2

Select all that apply

[X]	2.1. Waiver of written documentation of consent ***(complete Part 3)***.
[ ]	2.2. Waiver of consent or required elements of consent ***(complete Part 4)***.
[ ]	2.3. Use of an alternative method for documentation of consent, *i.e., "short form" consent* ***(complete Part 5)***.

3. Waiver of Written Documentation of Consent

Section 1: Description

PART 3 -SECTION 1

3.1.1. Briefly describe the procedures and/or subject populations for which consent will be obtained without written documentation of the consent (i.e., without a consent form). Do not refer to your IRB application.

We request waiver of documentation of written consent for the screening of potential participants with lower limb loss. We will screen potential participants over the telephone to assess their interest and eligibility for participation in the study. We will provide details about the study, including the purpose, the location and time commitment, the procedures, and the risks and benefits. We will then assess eligibility by asking their age, level and date of their amputation, if they are in good health, if they have wounds or sores on their residual limb, the prostheses that they own, if they can walk independently for six minutes, and if they are able to read and write in English. If they are eligible, we will then schedule an appointment for data collection. Written consent for all other study procedures will be obtained at their scheduled data collection session.

3.1.2. Indicate how you will provide the subjects with the required consent-related information about the research.

Select all that apply

[X]	An oral explanation of the research. *Examples: person-to-person, tape recording, or video recording*
[ ]	A written Information Sheet. *Examples: paper: in-person, faxed, mailed or electronic: email, website or webpage, text message, other*
[ ]	Other, describe below:

describe here

Section 2: Criteria for Approving Waiver

PART 3 -SECTION 2

3.2.1. Check the box next to the condition that best fits your study and then answer the questions for that condition. Provide specific details in your answers. *NOTE: the IRB cannot waive the requirement for documentation of consent for FDA-regulated research unless it meets Condition 1 below. The FDA does not accept Condition 2. Most research that involves giving a drug, device, supplement, botanical, or biologic to subjects is FDA-regulated.*

[X] CONDITION 1: Minimal risk

a. Does your research involve greater than minimal risk to the subjects?

"Research" means the specific procedures and subject groups for which you are requesting a waiver of consent. This may be only a portion of your research study.

"Minimal risk" means that the probability and magnitude of harm or discomfort anticipated in the research are not greater in and of themselves than those ordinarily encountered in daily life or during the performance of routine physical or psychological examinations.

[ ] **YES** → If "YES", a waiver of consent documentation cannot be granted under this Condition. *Look at the questions for Condition 2 to see if your research qualifies for a waiver of consent documentation under Condition 2.*

[X] **NO** → If "NO", justify your answer below:

The processes for which we request waiver of documentation of informed consent include asking potential participants 10 questions to assess study eligibility. Questions included in the telephone screen are general questions about age, amputation level, overall health, prosthetic use, etc. and are not likely to be perceived as invasive by respondents.

b. Does your research involve any procedures for which written consent is normally required outside of the research context? *Examples: in everyday life, written consent is needed for surgery but not for many surveys or for non-invasive health measurements by your health care provider.*

[X] **NO**

[ ] **YES** → If "YES", a waiver of consent documentation cannot be granted under this Condition. *Look at the questions for Condition 2 to see if your research qualifies for a waiver of consent documentation under Condition 2.*

[ ] CONDITION 2: Signed consent document is the primary risk

c. Is the existence of a signed consent document the only document or record that would link the subject to the research? (This means that data and specimens will not be recorded or stored with identifiers or links to identifiers.)

[ ] **NO** → If "NO", a waiver of consent documentation cannot be granted under this Condition. *Look at the questions for Condition 1 to see if your research qualifies for a waiver of consent documentation under Condition 1.*

[ ] **YES**

d. Is the principal risk associated with the research the potential harm to subjects that might occur if there was a breach of confidentiality about their participation? *Example: a study that involves subjects who use illegal drugs.*

PART 3 - SECTION 2

[ ] **NO**

[ ] **YES** → If "YES", justify your answer below:

justify here

e. Will subjects be asked whether they want documentation linking them with the research, and the subjects' wishes will govern?

[ ] **YES**

[ ] **NO** → If "NO", a waiver of consent documentation cannot be granted under this Condition. *Look at the questions for Condition 1 to see if your research qualifies for a waiver of consent documentation under Condition 1.*

4. Waiver of Consent Waiver/Alteration of Elements of Consent

Section 1: Basic Eligibility

PART 4 SECTION 1

4.1.1. Is your research regulated by the FDA (Food and Drug Administration)? *Most research that involves a drug, device, supplement, biologic, or botanical is FDA-regulated. If you are not sure, please contact the Human Subjects Division for assistance before completing this form. Email: hsdinfo@u.washington.edu Phone: 206-543-0098*

[ ] **NO**

[ ] **YES** → If "YES", is your request for a waiver of consent ONLY for the activity of screening (or "pre" screening) records to identify possible subjects for the research?

[ ] **NO** → If "NO", do not complete this form. FDA regulations do not allow a waiver of consent for any research activities except specific types of emergency medicine research or treatment. If you are planning emergency medicine research, contact the Human Subjects Division for assistance. Email: hsdinfo@u.washington.edu Phone: 206-543-0098.

[ ] **YES** → If "YES", complete this form.

Section 2: Description

PART 4 -SECTION 2

4.2.1. Which of the following are you requesting?

Select all that apply

[ ] A waiver of the requirement to obtain consent.

[ ] A waiver or alteration of one or more of the specific required elements of consent. See Section 3.3 of the SOP Consent for more information.

4.2.2. Briefly describe the procedures and/or subject populations for which you are requesting a waiver of consent or partial waiver/alteration of consent. Do not refer to your IRB application.

describe here

Section 3: Criteria for Approving Waiver

PART 4 - SECTION 3

4.3.1. Check the box next to the condition that best fits your study and then answer the questions for that condition. Provide specific details in your answers.

[ ] CONDITION 1: Minimal risk

PART 4 -SECTION 3

a. Does the research involve greater than minimal risk to the subjects?

"Research" means the specific procedures and subject groups for which you are requesting a waiver of consent. This may be only a portion of your research study.

"Minimal risk" means that the probability and magnitude of harm or discomfort anticipated in the research are not greater in and of themselves than those ordinarily encountered in daily life or during the performance of routine physical or psychological examinations.

[ ] **YES** → If "YES", the waiver of consent cannot be granted under this Condition. *Look at the questions for Condition 2 to see if your research qualifies for a waiver of consent under Condition 2.*

[ ] **NO** → If "NO", justify your answer below:

justify here

b. Will a waiver of consent adversely affect the rights and welfare of the subjects?

[ ] **YES** → If "YES", a waiver of consent cannot be granted under this Condition. *Look at the questions for Condition 2 to see if your research qualifies for a waiver of consent under Condition 2.*

[ ] **NO** → If "NO", justify your answer below:

justify here

c. Could the research practicably be carried out without the waiver of consent?

[ ] **YES** → If "YES", a waiver of consent cannot be granted under this Condition. *Look at the questions for Condition 2 to see if your research qualifies for a waiver of consent under Condition 2.*

[ ] **NO** → If "NO", select from the reasons below:

Select all that apply.

[ ] It is not possible to contact all of the subjects associated with the data or specimens in order to obtain consent.

[ ] The design of the study does not allow the possibility of obtaining consent.

[ ] The potential study population is so large that it would not be feasible to obtain consent.

[ ] The research cannot be conducted with a population for whom consent could practicably be carried out.

[ ] Alternative methods for obtaining consent (for example, consent over the phone) are not feasible.

[ ] Requiring informed consent may introduce systematic bias into the data.

[ ] The risk of contacting the subjects is greater than the risk of the study procedures.

[ ] Other, please describe:

describe here

Provide information supporting your answer, to assist the IRB in understanding why obtaining consent or the indicated elements of consent would not be feasible.

justify your answer here

d. Would it be appropriate to provide subjects with information about the study after their participation? *This is not required in order to grant the waiver. However, answering the question IS a requirement for granting the waiver.*

Select all that apply:

- [ ] No, because the data will not be stored with, or linked to, identifiers.
- [ ] No, because the information that is found will have no impact on subjects' clinical care.
- [ ] No, because there is no feasible mechanism by which to notify subjects.
- [ ] No, because of another reason. Explain:

explain here

- [ ] Yes. Describe the information that will be provided and the procedures used to provide it:

describe here

[ ] CONDITION 2: State or local public benefit or service programs. *NOTE: This Condition is rarely applicable.*

a. Is the research to be conducted by, or subject to the approval of, state or local government officials?

[ ] **NO** → If "NO", a waiver of consent cannot be granted under this Condition. *Look at the questions for Condition 1 to see if your research qualifies for a waiver of consent under Condition 1. If it does not, then you do not need to submit this form unless you are requesting another type of consent waiver.*

[ ] **YES**

b. Is the research designed to study, evaluate or otherwise examine any of the following?

Select all that apply

- [ ] Public benefit or service programs. *Examples: unemployment benefits; state-funded health insurance.*
- [ ] Procedures for obtaining benefits or services under those programs.
- [ ] Possible changes in or alterations to those programs or procedures.
- [ ] Possible changes in methods or levels of payment for benefits or services under those programs.
- [ ] None of the above. → If "None of the above", a waiver of consent cannot be granted under this condition.

c. Could the research practicably be carried out without the waiver of consent?

[ ] **YES** → If "YES", a waiver of consent cannot be granted under this condition.

[ ] **NO** → If "NO", select from the reasons below:

Select all that apply

- [ ] It is not possible to contact all of the subjects associated with the data or specimens in order to obtain consent.
- [ ] The research cannot be conducted with a population for whom consent could practicably be carried out.
- [ ] Alternative methods for obtaining consent (for example, consent over the phone) are not feasible.
- [ ] The design of the study does not allow the possibility of obtaining consent.
- [ ] The potential study population is so large that it would not be feasible to obtain consent.

PART 4 –SECTION 3

[ ] Requiring informed consent may introduce systematic bias into the data.

[ ] The risk of contacting the subjects is greater than the risk of the study procedures.

[ ] Other, please describe:

describe here

Provide information supporting your answers, to assist the IRB in understanding why obtaining consent or the indicated elements of consent would not be feasible.

justify your answer here

Part 5. Short Form Consent - an alternate process for documenting consent

Section 1: Description

PART 5 – SECTION 1

5.1.1. Briefly describe the procedures and/or subject populations for which you would like to use the Short Form consent to document the consent process. Do not refer to your IRB application.

describe here

Section 2: Criteria for approving the use of a Short Form Consent

PART 5 - SECTION 2

5.2.1. Confirm that your research meets each of the following requirements by checking the appropriate boxes and answering the questions. If one or more of the statements does not apply to your research, you cannot use the Short Form consent process for documenting consent.

5.2.1.a. There is a **Short Form** written consent document in the subject's language, stating that (1) the required elements of consent have been presented orally to the subject or subject's representative; and (2) the subject agrees to take part in the research as described.

[ ] **YES**

5.2.1.b. All of the required elements of consent are presented orally to the subject or subject's representative, in the subject's language. *This may or may not require the use of a translator.*

[ ] **NO, and I am requesting a waiver for some elements of consent.** ***Complete Part 4, "Waiver of Consent or Elements of Consent" above.***

[ ] **YES**

5.2.1.c. There will be a witness to the oral presentation who is fluent in both English and the subject's language.

When the person obtaining consent is assisted by a translator, the translator may serve as the witness.

[ ] **YES** → Describe the individual(s) who will serve as the witness, including their relationship (if any) to the subjects and to the research study team.

describe here

5.2.1.d. There is a **Written Summary** of what is to be said to the subject or subject's representative. *If there will be a standard consent form in English (for example, for the literate subjects in the study), the standard consent form can be used as the Written Summary if a Witness Signature Line is added to it.*

[ ] **NO**

[ ] **YES**

PART 5 – SECTION 2

5.2.1.e. Confirm by marking the box below that signatures will be obtained as described below:

[ ] **YES**

Short Form Signatures (can be X or mark)
- Subject or subject's representative
- Witness

Written Summary Signatures
- Witness (can be X or mark)
- Person obtaining consent

5.2.1.f. Confirm by marking the box below that copies will be given as described below:

[ ] **YES**

Short Form Received By
- Subject or subject's representative
- Researcher and/or study files

Written Summary Received By
- Subject or subject's representative
- Researcher and/or study files

5.2.2. Do you expect your research to include subjects who do not speak English?

[ ] **NO**

[ ] **YES** → If "YES", confirm that your research meets each of the following requirements for use of the Short Form consent process with subjects who do not speak English, by checking the appropriate boxes. If one or more of the statements does not apply to your research, you cannot use the short form consent process for documenting consent.

5.2.2.a. The witness will be someone who is fluent in both English and the language of the subject.

[ ] **YES**

5.2.2.b. The IRB-approved English language consent form will serve as the written summary. *(See part d of Question 5.2.1, above).*

[ ] **YES**

5.2.2.c. The oral presentation and the short consent form will be in a language understandable to the subject.

[ ] **YES**

5.2.2.d. The IRB will receive all foreign language versions of the short consent form. This is a condition for receiving IRB approval of this research.

[ ] **YES**

UNIVERSITY of WASHINGTON
HUMAN SUBJECTS DIVISION
Box 359470
Seattle, WA 98195-9470
Phone: 206-543-0098
Fax: 206-543-9218

APPLICATION: Modification, Approved Project

FOR HSD OFFICE USE ONLY

☐ MASTER COPY
☐ IRB WORKING COPY
☐ RESEARCHER COPY
☐ FULL IRB REVIEW REQUIRED
☐ EXPEDITED REVIEW

☐ APPROVED
☐ CONDITIONAL APPROVAL
☐ NOTED
☐ DISAPPROVED
☐ WITHDRAWN

DATE RECEIVED STAMP:

DORA
MOD #:

DATE OF IRB ACTION:

PRINTED NAME:

IRB CHAIR OR DESIGNEE SIGNATURE:

NOTES:

1. Research Study & Contact Information

Full Application Title:	IRB #:	Committee:
Evaluation of a Modified Running Prosthesis	49150	E/B

Lead Researcher Information (change of lead researcher requires a modification)

Name:	Title:	Position (e.g. Assistant Professor or Director):
Brian J. Hafner, PhD		Associate Professor

Home Institution (or source of paycheck):
UW Student? Home Institution is UW.
University of Washington

UW Department:	UW Division (Department of Medicine):
Rehabilitation Medicine	Prosthetics & Orthotics

UW Position or Appointment of Lead Researcher (choose the most appropriate one):

◉ Faculty
- ◉ Regular Faculty Appointment
- ◯ Research Faculty Appointment
- ◯ Clinical Faculty Appointment
- ◯ Affiliate Faculty Appointment
- ◯ Visiting Faculty Appointment
- ◯ Dual Appointment with PNNL
- ◯ Other (Describe):

◯ Student

◯ UW Resident or Fellow
◯ UW Administration or Staff
◯ None

Phone #:	Campus Box #:	Email:	Other address if not at UW:
206-685-4048	356490	bhafner@uw.edu	

Contact Person for the IRB (Change of contact person requires a modification)

Name:	Title:	Position (e.g. Assistant Professor or Director):
Cody McDonald, CPO		Graduate Research Assistant

Home Institution (or source of paycheck):
University of Washington

UW Department:	UW Division (Department of Medicine):

Rehabilitation Medicine	Prosthetics & Orthotics

UW Position or Appointment of IRB Contact Person (choose the most appropriate one):

- ◯ Faculty
- ◉ Student — ◉ Graduate or Professional Student (Matriculated or Approved "On Leave") — ◯ Matriculated Undergraduate Student — ◯ WWAMI Student
- ◯ UW Resident or Fellow — ◯ UW Administration or Staff — ◯ None

Phone #:	Campus Box#:	Email:	Other address if not at UW:
206-221-6347	356490	codym@uw.edu	

Name and Mailing Address for all paper-based correspondence

(If blank, correspondence will be directed to contact person or lead researcher if no contact person.)

Name:	Campus Box #:	Other address if not at UW:
Brian Hafner	356490	

Name of person completing this form (if not Lead Researcher or IRB Contact)

Name:	Email:	Phone:

END PART ONE

2. Reason(s) Submitted

☒ Researcher or Sponsor Initiated Modification *(Check all the types of modifications you are requesting. The requested sections will then be available in the form.)*:

- ☐ Part 3: Purpose
- ☐ Part 11: Waiver of HIPAA Authorization
- ☒ Part 4: Procedures
- ☐ Part 12: UW Confidentiality Agreement
- ☐ Part 5: Populations
- ☐ Part 13: Researchers and Research Staff
- ☒ Part 6: Recruitment
- ☐ Part 14: Individuals Performing Research Procedures
- ☒ Part 7: Consent/Assent
- ☐ Part 15: Non-UW Individuals, Organizations and Locations
- ☐ Part 8: Waiver of Documentation of Consent
- ☐ Part 16: Investigator Brochure and/or Protocol Amendments
- ☐ Part 9: Waiver of Consent or Waiver/Alteration of Elements of Consent
- ☐ Part 17: Funding
- ☐ Part 10: Confidentiality of Research Data
- ☐ Part 18: Other Compliance Approval Letters/ Reports (Radiation Safety Approval, Data Safety Monitoring Reports)

END PART TWO

4. Procedures

PURPOSE OF THIS SECTION: Complete this section if you intend to change any research procedures. Procedures include (but are not limited to) developing research study instruments, changing your recruitment process, changing your consent process, requesting review of medical or other records for pre-screening or requesting records review as part of your research, changing the amount of compensation offered to subjects, adding additional surveys, questionnaires or interventions.

4.1. Summarize the proposed changes or new procedures:

We will add another study session to the study protocol where we will assign participants' first prostheses and to attach an activity monitor to the selected prosthesis. Previously, we were going to ask participants to do this step on their own.

4.2. Explain why the changes are being made:

The activity monitor we provided to study participants needs to be positioned correctly on the prosthesis to collect

valid data. Participants may be challenged to attach it correctly or lose it if we give it to them. The new session is added so that study investigators can properly attach the activity monitor to the participants' prosthesis. Additionally, we will ask participants to leave one of their study prostheses with us for the duration of the study. This is important because the data we collect may be confounded if participants wear both prostheses. Participants will be paid for their time and their parking will be reimbursed for this extra session. No other procedures are changed.

4.3. Do the changes affect any of the following processes and/or documents?

4.3.a. Recruitment process and documents, including advertisements?

- (•) **YES** Complete Part 6
- ( ) **NO**

4.3.b. Consent process and documents?

- (•) **YES** Complete Part 7
- ( ) **NO**

4.3.c. Records and/or research data?

- ( ) **YES**
- (•) **NO**

4.3.d. Radiation exposure?

- ( ) **YES**
- (•) **NO**
- ( ) **N/A**

4.3.e. Administrative/Other?

- ( ) **YES**
- (•) **NO**

4.4. Describe the effects of these changes on the risks and/or benefits to subjects. If there are no changes in the risks or benefits, state: "None".

None

4.5. Are you adding the use of a drug, medical device, biologic, botanical, or dietary supplement? For more information see the SOP FDA-Regulated Research.

- ( ) **YES**
- (•) **NO**

4.6. Are you adding any procedures that involve genetic research?

Genetic research is defined as research involving the analysis of any of the following: DNA; RNA; chromosomes; mitochondria; any or all parts of the human genome; or biomarkers such as proteins or metabolites which may be implicated in, associated with, or cosegregated with a disorder, syndrome, condition, or predisposition to disease or behavior. Usually genetic research involves the collection and/or use of human biological specimens such as blood, skin, or other tissues, nail clippings, or hair. Genetic research may also include the construction of pedigrees ("maps" of the distribution of a particular trait or condition among related individuals) or family medical histories.

- ( ) **YES**
- (•) **NO**

4.7. Are you adding any procedures that involve any component of the federal Department of Defense (DOD)? For more information see the SOP Department of Defense.

- ( ) **YES**
- (•) **NO**

4.8. Are you adding any procedures that involves the federal Department of Justice (DOJ) or any of its components (such as the National Institute of Justice, or any facilities/personnel of the Bureau of Prisons)? For more information see the SOP Department of Justice Research.

- ( ) **YES**
- (•) **NO**

4.9. Are you adding any procedures that take place under, or otherwise involve, general anesthesia?

- ( ) **YES**
- (•) **NO**

END PART FOUR

BACK TO TOP

6. Recruitment

6.1. Provide a general overview and context for the changes you are making to recruitment:

The telephone screening script and recruitment flyer have been revised to reflect addition of the extra session. In addition, we have added an screening (and inclusion) criteria to the telephone script that indicates participants must be willing to leave one prosthesis with us for the duration of the study . They will always have one prosthesis to wear, as they would normally if they were not part of this study.

6.2. Describe the proposed changes to your recruitment process:
- If there are multiple recruitment strategies, describe each one individually;
- If there are multiple subject groups and your recruitment differs for each, describe the recruitment for each group.

The recruitment process is unchanged from our original application.

6.3. Explain who will approach subjects and how this will be done to protect subjects' privacy:

Individuals interested in participation will be directed by the posted study flyer to contact the investigators for information and/or screening.

6.4. Describe how you will minimize potential coercion or undue influence during recruitment:

Individuals interested in participation will not be recruited by their health care provider (Mr. Davidson of Davidson Prosthetics). Mr. Davidson will only inform potential participants about the study through posting (or provision) of an informational study flyer. All recruitment/enrollment procedures will be conducted by study investigators/staff. Interested individuals will be informed their participation in the study is voluntary and in no way affects the care they will received from their provider. No members of our study team are or will be involved in the clinical care of potential participants or participants.

6.5. Describe any changes to subjects gifts, payments, services without charge, or extra course credit, and include the value/ dollar amount, if applicable:

Participants will be paid $30/hr for the extra session. Parking will also be paid/reimbursed. The screening script and flyer have been updated to include this information.

6.6. Complete the table below for each new or revised recruitment document.
- Identify the documents that are being revised and the documents that are new.
- Attach all of the materials in the same order that you listed the documents.
- Submit 3 clean copies and 3 copies with the revisions in tracked changes (2 copies for Minimal Risk).

Examples for Row #1 - "Type of recruitment materials" can be:

- Advertisement
- Email
- Flyer
- Letter to subjects
- Letter to colleagues
- Magazine ad or article
- Newspaper ad or article
- Oral script
- Poster
- Radio ad
- Television ad
- Website text and layout

Examples for Row #2 - "Reason submitted" can be:

- Adding new recruitment flyer
- Deleting old recruitment letter to subjects, no longer in use
- Revising existing oral script
- Replacing existing oral script with new one

END PART SIX - Also complete the "recruitment materials" table(s)

BACK TO TOP

Recruitment materials		Table # 1
**Row 1**	Type of recruitment document or material:	Telephone Screening Script
**Row 2**	Reason submitted:	Updated to reflect the revised, 4-visit protocol and revised inclusion/exclusion criteria (several criteria on the Telephone screening script incorrectly identified required answer for eligibility. Questions were improved for clarity and grammar.)

**Row 3**	IF APPLICABLE<br>IRB Approval Date:<br>(most recent approval date of recruiting material being revised or replaced.)	02/12/2015

Recruitment materials		Table # 2
**Row 1**	Type of recruitment document or material:	Study Flyer
**Row 2**	Reason submitted:	Updated to reflect revised 4-visit protocol and enhanced for visual appearance.
**Row 3**	IF APPLICABLE<br>IRB Approval Date:<br>(most recent approval date of recruiting material being revised or replaced.)	02/12/2015

Add Materials Table

Remove Materials Table

7. Consent/Assent

7.1. Provide a general overview and context for the changes you are making: (For more information on Consent and Consent Documentation, see the SOPs linked here.)

The consent form has been revised to reflect addition of the extra session. In addition, we have added information that indicates participants must be willing to leave one prosthesis with us for the duration of the study. They will always have one prosthesis to wear, as they would normally if they were not part of this study.

7.2. Describe the proposed changes to your consent process(es), and/or describe any new consent process(es) being added.
- If there are multiple new or revised consent processes, describe each one individually.
- Include information about who obtains consent, when, and how.

Participants will be informed in the consent form of the expectation that they leave one prosthesis with us during the period of study.

7.3. Do you plan to re-consent subjects?

(●) **YES** Describe your re-consenting process:

We have not yet recruited study participants. Any participants recruited under older processes will be re-consented at their next study visit.

( ) **NO**

7.4. Provide a complete list of all new or revised consent materials by completing the table(s) below for each new or revised consent document:
- Identify the documents that are being revised and the documents that are new.
- If you are only submitting new consent documents, write “Not applicable” in the table below, where appropriate.
- Attach all of the consent materials in the same order that you listed the documents.
- Submit 3 clean copies and 3 copies with the revisions in tracked changes. (2 copies for Minimal Risk.)

Examples for Row #1 - "Type of consent materials" can be:

- Consent Form
- Parent Consent Form
- Parent Consent/Assent Form
- Assent Form for Age 0-6, Assent Form for Age 7-12, or Assent Form for Age 13-17
- Oral Consent Script
- Information Sheet
- Translated Consent Form
- Back-Translated Consent Form
- Consent Form approved by another IRB
- Sub-study Consent Form

Examples for Row #2 - "Reason submitted" can be:

- Adding new consent form
- Adding new addendum to consent form
- Deleting old consent form
- Revising existing consent form to update research staff
- Replacing existing consent form that is outdated

END PART SEVEN - Also complete the "consent materials" table(s) BACK TO TOP

Consent materials		Table # 1	
**Row 1**	Type of consent document or material:	Consent form	
**Row 2**	Reason submitted:	Revised existing telephone screening script and consent forms.	
**Row 3**	Consent Form Title:	Evaluation of a Modified Running Prosthesis	Evaluation of a Modified Running Prosthesis
**Row 4**	Version number and/or revision date:	02/14/2015	04/24/2015
**Row 5**	IF APPLICABLE Consent Form Footer:	02/12/2015	04/24/2015
**Row 6**	IF APPLICABLE IRB Approval Date: (most recent approval date of consent form being revised or replaced)	02/19/2015	

Add Materials Table

Remove Materials Table

19. Attachments

- Check to make sure that all of the required attachments are included with this submission.
- Collate all of your attachments.
- Use clips, not staples, with at least one packet, so that HSD staff may easily distribute your materials to additional IRB reviewers as needed.
- If you are attaching **consent forms and materials and/or recruitment materials, provide a total of 3 clean copies of each document and a total of 3 copies of each document with the revisions in "tracked changes". (2 copies for minimal risk)**
- Unless otherwise instructed below, include 3 copies of each document. (2 copies for minimal risk)
- When possible, please order your documents as listed below.
- You should have a total of 3 complete submission "packets" with attachments included. (2 copies for minimal risk)

Explanation of Attachments (if necessary):

- ☐ Assent form(s)
- ☐ Confidentiality Agreement ***(1 original ink-signed copy ONLY)***
- ☒ Consent form(s)
- ☐ Consent materials translated into a language other than English
- ☐ Consent Materials: addendum consent, information sheets, oral consent scripts
- ☐ Data collection instruments/forms
- ☐ Data safety and monitoring charter and/or report(s)
- ☐ Data Safety Monitoring Plan
- ☐ Data Use Agreement(s)
- ☐ Department of Anesthesiology Approval
- ☐ Embryonic Stem Cell Research Oversight Committee (ESCRO) approvals/letters/report
- ☐ Engagement Worksheet
- ☐ Environmental Health and Safety (EHS) approvals/letters/report
- ☐ Federal Certificate of Confidentiality or Privacy Certificate
- ☐ Grant application and title page of grant application ***(1 copy ONLY)***
- ☐ Implant and Investigational Device Committee (IIDC) approvals/letters/report
- ☐ Individual Investigator Agreements
- ☐ Institutional Biosafety Committee (IBC) approvals/letters/report
- ☐ Investigator brochure ***(1 copy ONLY)***
- ☐ IRB Authorization Agreements
- ☐ Literature or abstracts supporting the purpose of your research
- ☐ Material Transfer Agreement(s) (MTA)
- ☐ Oral scripts
- ☐ Other funding documentation, only if you have funding that is not a grant application/proposal
- ☐ Non-UW IRB approval letters/notifications
- ☐ Non-UW IRB approved applications
- ☐ Protocol ***(1 copy ONLY)***
- ☐ Radiation Safety Applications or Radiation Safety Approval Letters (RS)
- ☐ Radioactive Drug Research Committee (RDRC) approvals/letters/report
- ☐ Recruitment - electronic materials: scripts for emails, and/or copies of web pages
- ☒ Recruitment - oral materials: scripts, radio ads
- ☒ Recruitment - written materials: flyers, brochures, newspaper ads, and/or letters
- ☐ Study instruments: surveys, questionnaires, assessment tools, tracking forms, web surveys
- ☐ SUPPLEMENT: Department of Defense Involvement
- ☐ SUPPLEMENT: Department of Justice
- ☐ SUPPLEMENT: Devices
- ☐ SUPPLEMENT: Drugs, Biologics, Botanicals
- ☐ SUPPLEMENT: Genetic Research
- ☐ SUPPLEMENT: GWAS dbGaP
- ☐ SUPPLEMENT: Protected/Vulnerable Populations
- ☐ SUPPLEMENT: Waiver Request, Consent Requirements
- ☐ SUPPLEMENT: Waiver Request, HIPAA Authorization
- ☐ Other, specify:

END ATTACHMENTS

BACK TO TOP

Print Form

UNIVERSITY of WASHINGTON
HUMAN SUBJECTS DIVISION
Box 359470
Seattle, WA 98195-9470
Phone: 206-543-0098
Fax: 206-543-9218

APPLICATION: Modification, Approved Project

FOR HSD OFFICE USE ONLY

DATE RECEIVED STAMP:

- ☐ MASTER COPY
- ☐ IRB WORKING COPY
- ☐ RESEARCHER COPY
- ☐ FULL IRB REVIEW REQUIRED
- ☐ EXPEDITED REVIEW
- ☐ APPROVED
- ☐ CONDITIONAL APPROVAL
- ☐ NOTED
- ☐ DISAPPROVED
- ☐ WITHDRAWN

DORA
MOD #:

DATE OF IRB ACTION:

PRINTED NAME:

IRB CHAIR OR DESIGNEE SIGNATURE:

NOTES:

1. Research Study & Contact Information

Full Application Title:	IRB #:	Committee:
Evaluation of a Modified Running Prosthesis	49150	E/B

Lead Researcher Information (change of lead researcher requires a modification)

Name:	Title:	Position (e.g. Assistant Professor or Director):
Brian J. Hafner, PhD		Associate Professor

Home Institution (or source of paycheck):
UW Student? Home Institution is UW.
University of Washington

UW Department:	UW Division (Department of Medicine):
Rehabilitation Medicine	Prosthetics & Orthotics

UW Position or Appointment of Lead Researcher (choose the most appropriate one):

- (●) Faculty
  - (●) Regular Faculty Appointment
  - ( ) Research Faculty Appointment
  - ( ) Clinical Faculty Appointment
  - ( ) Affiliate Faculty Appointment
  - ( ) Visiting Faculty Appointment
  - ( ) Dual Appointment with PNNL
  - ( ) Other (Describe):
- ( ) Student
- ( ) UW Resident or Fellow
- ( ) UW Administration or Staff
- ( ) None

Phone #:	Campus Box #:	Email:	Other address if not at UW:
206-685-4048	356490	bhafner@uw.edu	

Contact Person for the IRB (Change of contact person requires a modification)

Name:	Title:	Position (e.g. Assistant Professor or Director):
Cody McDonald	CPO	Graduate Research Assistant

Home Institution (or source of paycheck):
University of Washington

UW Department:	UW Division (Department of Medicine):

Rehabilitation Medicine	Prosthetics & Orthotics

UW Position or Appointment of IRB Contact Person (choose the most appropriate one):

- ○ Faculty
- ◉ Student — ◉ Graduate or Professional Student (Matriculated or Approved "On Leave") ○ Matriculated Undergraduate Student ○ WWAMI Student
- ○ UW Resident or Fellow ○ UW Administration or Staff ○ None

Phone #:	Campus Box#:	Email:	Other address if not at UW:
206-221-6347	356490	codym@uw.edu	

Name and Mailing Address for all paper-based correspondence
(If blank, correspondence will be directed to contact person or lead researcher if no contact person.)

Name:	Campus Box #:	Other address if not at UW:
Brian Hafner	356490	

Name of person completing this form (if not Lead Researcher or IRB Contact)

Name:	Email:	Phone:

END PART ONE

2. Reason(s) Submitted

☒ Researcher or Sponsor Initiated Modification *(Check all the types of modifications you are requesting. The requested sections will then be available in the form.)*:

- ☐ Part 3: Purpose
- ☐ Part 4: Procedures
- ☒ Part 5: Populations
- ☐ Part 6: Recruitment
- ☒ Part 7: Consent/Assent
- ☐ Part 8: Waiver of Documentation of Consent
- ☐ Part 9: Waiver of Consent or Waiver/Alteration of Elements of Consent
- ☐ Part 10: Confidentiality of Research Data
- ☐ Part 11: Waiver of HIPAA Authorization
- ☐ Part 12: UW Confidentiality Agreement
- ☒ Part 13: Researchers and Research Staff
- ☐ Part 14: Individuals Performing Research Procedures
- ☐ Part 15: Non-UW Individuals, Organizations and Locations
- ☐ Part 16: Investigator Brochure and/or Protocol Amendments
- ☒ Part 17: Funding
- ☐ Part 18: Other Compliance Approval Letters/ Reports (Radiation Safety Approval, Data Safety Monitoring Reports)

END PART TWO

5. Populations

5.1. Identify the changes you are requesting in subject population(s).

- ☐ 5.1.a. Add new subject population(s).
- ☐ 5.1.b. Change eligibility criteria of already-approved study population(s).
- ☒ 5.1.c. Change number of subjects approved to complete the study for an already-approved study population.
- ☐ 5.1.d. Remove existing study population(s).

5.2. Briefly describe the proposed changes in subjects population(s), and explain the reasons for this change:

We will change the desired sample from 24 to 60 individuals. All inclusion/exclusion criteria remain the same. The reason for this change is new funding that will allow us to recruit additional participants.

5.2.a. Describe changes (if any) to the following for each already-approved study population, and describe the following for any new study populations being added:

Inclusion Criteria:	No changes
Exclusion Criteria:	No changes
Age Range:	No changes
Number of Subjects:	60

5.3. Do the changes affect the consent form(s)?

○ YES

◉ NO — Explain why not:

No changes are made to study procedures or data collection processes.

5.7. Do the changes involve adding a population of subjects (or records) from the Department of Defense (DOD)? For more information see the SOP Department of Defense.

○ YES

◉ NO

END PART FIVE

BACK TO TOP

7. Consent/Assent

7.1. Provide a general overview and context for the changes you are making: (For more information on Consent and Consent Documentation, see the SOPs linked here.)

The consent form has been revised to reflect the additional funding source and new study investigator.

7.2. Describe the proposed changes to your consent process(es), and/or describe any new consent process(es) being added.
- If there are multiple new or revised consent processes, describe each one individually.
- Include information about who obtains consent, when, and how.

No changes will be made to consent processes.

7.3. Do you plan to re-consent subjects?

◉ YES — Describe your re-consenting process:

Participants consented with the previous form will be re-consented at their next study visit.

○ NO

7.4. Provide a complete list of all new or revised consent materials by completing the table(s) below for each new or revised consent document:
- Identify the documents that are being revised and the documents that are new.
- If you are only submitting new consent documents, write “Not applicable” in the table below, where appropriate.
- Attach all of the consent materials in the same order that you listed the documents.
- Submit 3 clean copies and 3 copies with the revisions in tracked changes. (2 copies for Minimal Risk.)

Examples for Row #1 - "Type of consent materials" can be:

- Consent Form
- Parent Consent Form
- Parent Consent/Assent Form
- Assent Form for Age 0-6, Assent Form for Age 7-12, or Assent Form for Age 13-17
- Oral Consent Script
- Information Sheet
- Translated Consent Form
- Back-Translated Consent Form
- Consent Form approved by another IRB
- Sub-study Consent Form

Examples for Row #2 - "Reason submitted" can be:

- Adding new consent form
- Adding new addendum to consent form
- Deleting old consent form
- Revising existing consent form to update research staff
- Replacing existing consent form that is outdated

END PART SEVEN - Also complete the "consent materials" table(s)

BACK TO TOP

Consent materials — Table # 1

**Row 1**	Type of consent document or material:	Consent form	
**Row 2**	Reason submitted:	Additional funding source and study investigator added.	
**Row 3**	Consent Form Title:	Evaluation of a Modified Running Prosthesis	Evaluation of a Modified Running Prosthesis
**Row 4**	Version number and/or revision date:	05/27/2015	06/03/2015
**Row 5**	IF APPLICABLE Consent Form Footer:	05/27/2015	06/03/2015
**Row 6**	IF APPLICABLE IRB Approval Date: (most recent approval date of consent form being revised or replaced)	06/01/2015	

Add Materials Table

Remove Materials Table

13. Researchers and Research Staff

Complete Part 13 when you are changing the lead researcher, study coordinator, IRB contact person, subject contact person, faculty advisor.

- Select "ADD" to add another study coordinator, IRB contact person, subject contact person to your project.
- Select "CHANGE" to replace the current lead researcher, study coordinator, IRB contact person, subject contact person, faculty advisor on your project.
- Select "REMOVE" to remove, without replacing, a study coordinator, IRB contact person, subject contact person from your project.

NOTE: Lead Researchers and Faculty Advisors can only be CHANGED.

FOR MORE INFORMATION ON WHO NEEDS TO BE LISTED ON THE IRB APPLICATION SEE: SOP Research Team

	ADD	CHANGE	REMOVE
13.1. Lead Researcher		○	
13.2. Study Coordinator	○	○	○
13.3. IRB Contact Person	○	○	○
Subject Contact Person #:1	ADD	CHANGE	REMOVE
13.4. Subject Contact Person	◉	○	○

b. Provide the following information for the new Subject Contact Person:

Name:	Title:	Position (e.g. Asst. Prof. or Director):
Sara Morgan, CPO, PhD		Acting Assistant Professor

Home Institution: University of Washington

UW Department:	UW Division (School of Medicine):
Rehabilitation Medicine	Prosthetics & Orthotics

UW Position or Appointment (choose the most appropriate one):

- ◉ Faculty
  - ◉ Standard Faculty Appointment
  - ○ Research Faculty Appointment
  - ○ Clinical Faculty Appointment
  - ○ Affiliate Faculty Appointment
  - ○ Visiting Faculty Appointment
  - ○ Dual Appt. with PNNL
  - ○ Other - Describe:
- ○ Student
- ○ UW Resident, Fellow, or Post-Doc at UW or Local VA
- ○ UW Administration or Staff
- ○ None

Phone	Campus Box #:	Email:	Other address if not at UW:
206-616-9997	356490	sjmorgan@uw.edu	

c. Reason why the Subject Contact Person is being added:

New investigator added to the study with additional funding.

IMPORTANT: Be sure to revise any documents that mention the Subject Contact Person's name, including consent forms and any Confidentiality Agreement. The revision should be submitted as part of this Modification. Any other aspects of the study (e.g. recruiting, study procedures) that will be affected by this change should also be submitted with this Modification.

Add, change, or remove another subject contact person | Cancel

	ADD	CHANGE	REMOVE
13.5. Faculty Advisor		○	

END PART THIRTEEN

BACK TO TOP

17. Funding

17.1. Select from the following options. (A and/or B)

☐	**A** Delete Funding
☒	**B** Add Awarded Funding

PART SEVENTEEN INCLUDES TWO SECTIONS

BACK TO TOP

Part 17: Section 2: Adding Funding or Support

17.2.1. Do the consent forms or the consenting process need to be modified? (It is UW policy that external funding must be listed on the consent form.)

◉ **YES** — Complete Part 7 in the Modification Form and include 3 clean copies of the consent form(s) and 3 copies of the consent form(s) with the revisions in tracked changes (2 copies for Minimal Risk.)

○ **NO**

17.2.2. Does anyone on the research team now have a financial conflict of interest with respect to this research (as defined by UW Policy GIM 10) because of this new funding?

○ **YES**

◉ **NO**

17.2.3. Submit 1 copy of the grant application, contract, or other type of funding proposal with the pages pertaining to the research study flagged.

- Include the title page of the grant proposal, if applicable
- If there are multiple aims associated with this new funding (e.g. a new grant, contract, subcontract, etc.), please specify which aim(s) are relevant to this specific IRB application below:

17.2.4. If adding funding or support will also change any of your research procedures, please also complete Part 4: Procedures

Complete the information in the "Funding or Support" box below for each new or revised (updated) funding source.

END PART SEVENTEEN SECTION TWO - Also complete the "funding or support" table(s)

Funding or Support — Table #1

Type of Support:
Include indirect federal support: generic (i.e., not tied to this specific study) federal salary support for the time that any key personnel spend on the research. Examples: Many training grants, fellowships, scholarships and career development awards.

☒ Grant	☐ Center Grant	☐ Training Grant	☐ University Funds
☐ Gift	☐ Fellowship	☐ Department Funds	☐ Contract
☐ Other, describe:			
☐ Subcontract			

Title of Grant, Contract or Award:	A Novel Prosthetic Foot Designed to Maximize Functional Abilities, Health Outcomes, and Quality of Life in People With Transtibial Amputation
PI on Grant, Contract or Award:	Brian J. Hafner, PhD
Funding Agency or Sponsor:	Dept. of the Army -- USAMRAA

If you are adding funding from the federal Department of Justice (DOJ) or any component (such as the National Institute of Justice): complete and attach the SUPPLEMENT: Department of Justice.

If you are adding funding from the federal Department of Defense (DOD) complete and attach the SUPPLEMENT: Department of Defense Involvement.

Award Number:	OP140079		
Funding dates: ☐ N/A	Start: Jun 1, 2015	End: May 31, 2017	

What institution or agency processed the funding proposal?

☒ UW Office of Sponsored Programs (OSP)	☐ UW Royalty Research Fund	☐ Seattle Institute for Biomedical and Clinical Research	☐ VA Boise
☐ Fred Hutchinson Cancer Research Center	☐ UW Development Office	☐ Puget Sound Blood Center	☐ VA Seattle/American Lake
☐ Public Health Seattle/ King County	☐ Other, describe:		

19. Attachments

- Check to make sure that all of the required attachments are included with this submission.
- Collate all of your attachments.
- Use clips, not staples, with at least one packet, so that HSD staff may easily distribute your materials to additional IRB reviewers as needed.
- If you are attaching **consent forms and materials and/or recruitment materials, provide a total of 3 clean copies of each document and a total of 3 copies of each document with the revisions in "tracked changes". (2 copies for minimal risk)**
- Unless otherwise instructed below, include 3 copies of each document. (2 copies for minimal risk)
- When possible, please order your documents as listed below.
- You should have a total of 3 complete submission "packets" with attachments included. (2 copies for minimal risk)

Explanation of Attachments (if necessary):

- ☐ Assent form(s)
- ☐ Confidentiality Agreement ***(1 original ink-signed copy ONLY)***
- ☒ Consent form(s)
- ☐ Consent materials translated into a language other than English
- ☐ Consent Materials: addendum consent, information sheets, oral consent scripts
- ☐ Data collection instruments/forms
- ☐ Data safety and monitoring charter and/or report(s)
- ☐ Data Safety Monitoring Plan
- ☐ Data Use Agreement(s)
- ☐ Department of Anesthesiology Approval
- ☐ Embryonic Stem Cell Research Oversight Committee (ESCRO) approvals/letters/report
- ☐ Engagement Worksheet
- ☐ Environmental Health and Safety (EHS) approvals/letters/report
- ☐ Federal Certificate of Confidentiality or Privacy Certificate
- ☒ Grant application and title page of grant application ***(1 copy ONLY)***
- ☐ Implant and Investigational Device Committee (IIDC) approvals/letters/report
- ☐ Individual Investigator Agreements
- ☐ Institutional Biosafety Committee (IBC) approvals/letters/report
- ☐ Investigator brochure ***(1 copy ONLY)***
- ☐ IRB Authorization Agreements
- ☐ Literature or abstracts supporting the purpose of your research
- ☐ Material Transfer Agreement(s) (MTA)
- ☐ Oral scripts
- ☐ Other funding documentation, only if you have funding that is not a grant application/proposal
- ☐ Non-UW IRB approval letters/notifications
- ☐ Non-UW IRB approved applications
- ☐ Protocol ***(1 copy ONLY)***
- ☐ Radiation Safety Applications or Radiation Safety Approval Letters (RS)
- ☐ Radioactive Drug Research Committee (RDRC) approvals/letters/report
- ☐ Recruitment - electronic materials: scripts for emails, and/or copies of web pages
- ☐ Recruitment - oral materials: scripts, radio ads
- ☐ Recruitment - written materials: flyers, brochures, newspaper ads, and/or letters
- ☐ Study instruments: surveys, questionnaires, assessment tools, tracking forms, web surveys
- ☒ SUPPLEMENT: Department of Defense Involvement
- ☐ SUPPLEMENT: Department of Justice
- ☐ SUPPLEMENT: Devices
- ☐ SUPPLEMENT: Drugs, Biologics, Botanicals
- ☐ SUPPLEMENT: Genetic Research
- ☐ SUPPLEMENT: GWAS dbGaP
- ☐ SUPPLEMENT: Protected/Vulnerable Populations
- ☐ SUPPLEMENT: Waiver Request, Consent Requirements
- ☐ SUPPLEMENT: Waiver Request, HIPAA Authorization
- ☐ Other, specify:

END ATTACHMENTS

BACK TO TOP

Print Form
